# Early Warning Signals of Ecological Transitions: Methods for Spatial Patterns

**DOI:** 10.1371/journal.pone.0092097

**Published:** 2014-03-21

**Authors:** Sonia Kéfi, Vishwesha Guttal, William A. Brock, Stephen R. Carpenter, Aaron M. Ellison, Valerie N. Livina, David A. Seekell, Marten Scheffer, Egbert H. van Nes, Vasilis Dakos

**Affiliations:** 1 Institut des Sciences de l'Evolution, CNRS, Université de Montpellier II, Montpellier, France; 2 Centre for Ecological Sciences, Indian Institute of Science, Bangalore, India; 3 Department of Economics, University of Wisconsin, Madison, Wisconsin, United States of America; 4 Department of Economics, University of Missouri, Columbia, Missouri, United States of America; 5 Center for Limnology, University of Wisconsin, Madison, Wisconsin, United States of America; 6 Harvard Forest, Harvard University, Petersham, Massachusetts, United States of America; 7 National Physical Laboratory, Hampton Road, Teddington, United Kingdom; 8 Department of Environmental Sciences, University of Virginia, Charlottesville, Virginia, United States of America; 9 Department of Aquatic Ecology and Water Quality Management, Wageningen University, Wageningen, The Netherlands; 10 Integrative Ecology Group, Estacion Biologica de Donana, Sevilla, Spain; Universitat Pompeu Fabra, Spain

## Abstract

A number of ecosystems can exhibit abrupt shifts between alternative stable states. Because of their important ecological and economic consequences, recent research has focused on devising early warning signals for anticipating such abrupt ecological transitions. In particular, theoretical studies show that changes in spatial characteristics of the system could provide early warnings of approaching transitions. However, the empirical validation of these indicators lag behind their theoretical developments. Here, we summarize a range of currently available spatial early warning signals, suggest potential null models to interpret their trends, and apply them to three simulated spatial data sets of systems undergoing an abrupt transition. In addition to providing a step-by-step methodology for applying these signals to spatial data sets, we propose a statistical toolbox that may be used to help detect approaching transitions in a wide range of spatial data. We hope that our methodology together with the computer codes will stimulate the application and testing of spatial early warning signals on real spatial data.

## Introduction

A range of ecosystems, from lakes and forests to rangelands and coral reefs, can exhibit multiple stable states [1]. In such ecosystems, abrupt shifts between ecological states may lead to ecological and economic losses. This happens when ecosystems reach a ‘tipping point’, at which they may rapidly reorganize into an alternative state with contrasting features. Such shifts have been documented not only in ecosystems, but also in a wide spectrum of complex systems including physiological systems, financial markets, and human societies [1]. However, the enormous complexity of such systems and the lack of detailed understanding of their underlying processes make it difficult to identify the points at which these systems may experience major changes. To circumvent this problem, recent research has focused on devising early warning signals of imminent transitions [2].

A number of early warning signals for ecological transitions has been proposed based on a phenomenon called ‘critical slowing down’ that generally occurs prior to a ‘bifurcation’ [3], [4]. The closer a system is to a bifurcation point, the longer time it takes to recover to its stable state upon any disturbance. Theoretical studies of ecological models suggest that either a direct measure of slow recovery rate [3]–[5] or its manifestations in the temporal and spatial dynamics of the system can potentially act as generic early warning signals of an impending transition [2], [6]–[8]. This phenomenon of slowing down is expected to occur before a broad range of transitions, including, but not limited to, the so-called ‘catastrophic shifts’ [9]. Catastrophic shifts are a particular case of transitions that are especially relevant because of their possible association with hysteresis and their lack of reversibility [1].

Generic early warning signals evaluated on time series have attracted a lot of attention in the literature [2]. However, recent theoretical studies suggest that for ecosystems that are not well-mixed (such as drylands, boreal wetlands, or heterogeneous habitats which host mobile predators), changes in spatial characteristics of the system could provide early warnings of approaching transitions as well [5], [10]–[13]. More generally, the spatial structure of ecosystems can provide information about the ecosystem degradation level [14]–[22]. Spatial information allows us to devise additional kinds of indicators thus adding to our arsenal of early warning signals. At the same time, well-resolved spatial data is becoming increasingly available at low cost due to improved technology (such as remote sensing).

Empirical verification of these indicators, however, has not been able to keep pace with the rapid growth in theoretical studies, and a number of recent studies question the ability and practical efficiency of these indicators to anticipate upcoming regime shifts in real systems [23], [24]. A few recent laboratory and field experiments [25]–[29], as well as analysis of climatic paleo-records [30], suggest that generic leading indicators (i.e. variance, skewness and autocorrelation at-lag-1) may indeed be detected in time series of real systems prior to transitions, but the empirical validation of the spatial indicators remains scarce [15], [17], [20], [21], [25], [31].

The discrepancy between theoretical developments and their empirical validation arises from a number of issues, such as the lack of sufficiently resolved and long term data, as well as the lack of a coherent methodological framework that outlines the steps and statistical tools necessary to detect those signals. For indicators based on time series, these issues have been addressed in a recent paper which provides a methodological guide to practitioners and managers to detect early warnings in time series [32] (see also http://www.early-warning-signals.org).

Here, we complement the previous work on detecting early warnings in time series data by providing a step-by-step methodology for detecting early warning signals in spatial data sets. We gather, for the first time, all the early warning signals proposed in the literature so far in a spatial context. We apply these metrics on model-generated data sets along a degradation gradient, and we discuss their interpretation based on a few potential null models. Our analysis mimicks a situation where an ecosystem would be degrading and where we would have access to several snapshots of an ecosystem's spatial structure taken over a period of time, or at different locations along a degradation gradient. We hope that our methodology together with the computer codes will stimulate testing and applications of spatial early warning signals on spatial data (R-code for the spatial analysis can be found at https://github.com/earlywarningtoolbox/spatial_warnings).

## Methods

### Spatial indicators

We first give a brief overview of the spatial early warning signals proposed in the literature so far. More details about the indicators and their precise mathematical formulation are provided in Appendix S1. Table 1 summarizes the spatial indicators and their expected trends along a degradation gradient.

**Table 1:** Early warning signals of transitions in spatial data.

Method/Indicator	Phenomenon			Expected trend	Ref.
	Rising memory	Rising variability	Patchiness		
Spatial correlation	x			increase	[5]
Return time	x			increase	[12]
Discrete Fourier Transform	x			spectral reddening	[13]
Spatial variance		x	x	increase	[10], [11]
Spatial skewness		x	x	peaks (see caption)	[11]
Patch-size distributions			x	change in shape of the dist.	[15]
Regular spotted patterns			x	change in patch shape	[22]
Power spectrum			x	spectral reddening	[43]

Leading spatial indicator, the primary underlying phenomenon, the expected trend along a degradation gradient, and the original references in which these were proposed. The trend of spatial skewness depends on the nature of test data: It can show a nonmonotonic behavior (thus, a peaking) for transition from a low density state to higher density state. For discrete data, it typically shows a monotonic behavior (increasing or decreasing depending on whether it is transitioning from fully covered to bare state or the other way transition).

#### Slowing-down based indicators: spatial correlation and spectral properties (DFT)

Due to increased recovery time to local equilibrium after a perturbation, neighboring units become more like each other when a system approaches a bifurcation point, i.e. they become increasingly correlated [5]. The increasing spatial coherence can be quantified by the spatial correlation function, or Moran's I, between ecological states separated by a certain distance. The near-neighbor spatial correlation, the analog of autocorrelation at lag 1 for time series, is calculated for the distance between nearest neighboring units of the system.

Spatial spectral properties change as the system approaches a tipping point [13]. To quantify spectral properties, we compute the Discrete Fourier Transform (DFT) that decomposes spatial data into components of sine and cosine waves of different wavenumbers [33]. A wavenumber can be thought of as a ‘spatial frequency’, or the number of times that a pattern is repeated in a unit of spatial length. In a spatial data set, periodicity is visualized as wavelength, which is inversely related to wavenumber (i.e., a small wavenumber corresponds to a large wavelength and vice-versa). As DFT is generally a complex number, we often plot the power spectrum (also 2D-periodogram) which is the magnitude of the complex DFT matrix (see Appendix S1 for details). Increased memory manifests itself as spectral reddening, i.e. spatial variation becomes increasingly concentrated at low wavenumbers [13]; in other words, long wavelength fluctuations become dominant prior to a transition [34].

Two metrics that help characterize spatial patterns for periodicity and directionality can be evaluated from the power spectrum. First, the radial-spectrum (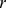-spectrum) is obtained by summing the power spectrum at constant distances from the origin of the power spectrum, i.e. along concentric circles at different distances from the center. It allows evaluating the periodicity of the patterns. Periodic patterns are characterized by a peak in the power spectrum. The wavenumber at which a peak occurs corresponds to the number of times that a pattern reproduces itself within a unit area of the spatial data, and therefore contains information about the scale of the pattern. Second, the angular-spectrum (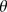-spectrum) is obtained by summing the values of the power spectrum using angular sectors. It allows evaluating the isotropy (orientation) of the spatial pattern. For an isotropic data set, the 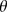-spectrum will show uniform amplitude at all angles, whereas for an anisotropic data set, the amplitude of the spectrum will show strong amplitudes for specific orientations [35].

#### Variability based indicators: Spatial variance and spatial skewness

Increased recovery time enroute to a bifurcation point may lead to stronger fluctuations around the equilibrium state of the system [36]. This can cause spatial variance of the system to increase prior to a transition [10], [11]. Spatial variance is formally defined as the second moment around the spatial mean of the state variable. It has also been shown that the fluctuations around the mean can become increasingly asymmetric as the system approaches a bifurcation point. This is because the fluctuations in the direction of the alternative stable state take longer to return back to the equilibrium than those in the opposite direction [11]; this asymmetry can also arise due to local flickering events (i.e. occasional jumps of local units between their current and alternative state) [37]. The spatial asymmetry can be measured by spatial skewness, which is the third central moment scaled by the standard deviation.

#### Patch based indicators: shapes and sizes of patches

Many ecological systems, such as shrublands in semi-arid ecosystems and mussel beds in the intertidal, exhibit striking spatial self-organized patterns [38]. It has been suggested that the nature of local ecological interactions, such as the relative scales of competition and facilitation, can strongly influence the type of emerging spatial patterns, leading to i) regular, periodic patches with a characteristic patch size [22], [38]–[40], or to ii) no characteristic scale of patchiness [15], [39]–[42]. These different types of spatial structures have been observed in a range of ecosystems, however their use as potential indicators of degradation has mostly been developed in the case of drylands, where both types of spatial structures exist. In drylands, it has been shown that the early warnings depend on the type of patchiness exhibited by the ecological system [12].

In ecosystems exhibiting periodic patterns, as the level of external stress increases, a predictable sequence of self-organized patterns based on ‘Turing instability’ occurs. In isotropic areas (i.e. no preferred orientation of the pattern) the shape of the patterns shifts from gaps to labyrinths and to spots as the system becomes more degraded. Thus, spotted vegetation patterns have been proposed to be an early warning signal of imminent desertification in drylands characterized by periodic patterns [16], [22]. In anisotropic areas with band-like patterns, the wavelength increases as the system approaches a transition [43].

In contrast to periodic patterns, there are cases where spatial processes give rise to non-periodic (irregular) patterns. In these cases, we can quantify the size of each patch and calculate the frequency of occurrence of different patch sizes. It is common practice to characterize the patchiness of these systems by a function that best describes the distribution of patch sizes. Irregular patterns may be characterized by a scale-free patch-size distribution, which means that there is no typical patch size in the ecosystem. Such a distribution may be well approximated by a pure power law [44] or by other heavy-tailed functions, such as a log-normal, a stretched exponential or a power law with cutoff [44], [45]. Scale-free patch-size distributions have been observed in several ecosystems [15], [20], [41], [42]. Computational models of drylands predict that larger vegetation patches become fragmented into smaller ones as aridity or grazing pressure increases and show an increasing deviation from a theoretical power law as the ecosystem approaches the desertification point [15], [46]. Therefore, it has been hypothesized that an increasing deviation from power-law distribution of patch sizes can signal increasing degradation (but see [17], [18]).

### Statistical significance tests

There are two steps in computing the spatial indicators. The first one is to compute the spatial metrics for a given snapshot. The second is to evaluate the trend of the spatial metrics along a degradation gradient (see Table 1). In doing so, we need to ensure that the spatial metrics for each snapshot and their trends differ from what would be expected by chance. A standard way to produce null models is to generate surrogate data and compare the trends in the indicators obtained from the original data to the trends obtained from the surrogate data [32]. Here, we discuss ways of obtaining null models for spatial early warning signals.

#### Null models

One way of obtaining a null model is to randomly permute or shuffle the elements of the spatial matrix, and this is also called bootstrapping. This is equivalent to a randomization procedure that removes any spatial structure from the original data but conserves the values of spatial variance and spatial skewness since these moments do not depend on the spatial arrangement of the data points. Therefore, such surrogate data cannot act as a null model for spatial variance or skewness but only for other metrics such as spatial autocorrelation, DFT, and patch-size distribution.

To devise a null model for spatial variance and skewness, Eby, Guttal and others (unpublished data) propose a coarse-graining method which should be applied for both the reshuffled matrix and the real data matrix. In this method, we first divide the full matrix of dimension 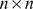 into nonoverlapping submatrices of size 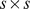. We then replace each submatrix by its average to obtain a smaller ‘coarse-grained matrix’ of size 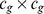 (note that 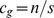). The basic intuition behind the method is as follows: consider any two non-overlapping submatrices of dimension (e.g. 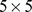) from the reshuffled matrix. Since the reshuffled matrix is equivalent to a random matrix, the average of the entries of the two sub-matrices chosen would be roughly equal to the average of the full matrix. This exercise of ‘coarse-graining’ necessarily reduces variability across submatrices in the case of a reshuffled (thus, random) matrix. Now, consider two non-overlapping submatrices in the real data. If we expect that the real data contains a non-random spatial pattern, the average of the entires of the two submatrices need not be of comparable value to each other nor with the average of the full real data matrix. Therefore, in contrast to the reshuffled matrix case, coarse-graining will not necessarily reduce variability in the real data, especially if it contains a spatial pattern. Since variability determines spatial variance and skewness, the coarse-graining applied to the reshuffled matrix provides a null model for spatial variance and spatial skewness.

An alternative method of building null models is to construct a spatial matrix from a continuous stochastic process. This method is applicable when continuous data (such as biomass density) is available at each spatial point as in data set 1 and 3. More specifically, we construct a null model matrix where each entry is a random number (e.g., from a normal distribution) whose mean and variance are equal to the mean and variance of the original data matrix, respectively. This approach provides a null model for spatial skewness, correlation, and DFT, but not for spatial variance since variance is, by construction, fixed to be the same as the one from the original matrix (see figures in Appendix S3). However, it may be claimed, following the same arguments as above, that the coarse-graining method can help estimate statistical significance of spatial variance.

#### The particular case of patchy ecosystems

A system with patchiness may show characteristic scales in the 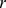
- and 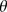-spectra. These 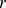
- and 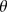-spectra of the data can be compared to those obtained from a null model. In the case where there is no spatial structure, it is known analytically that the values of the scaled power spectrum observed in each bin when calculating the 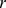
- or 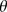-spectrum should be distributed as 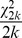 with 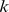 the number of values in the bin (see for instance [35], [47]; this gives very similar results as the confidence intervals presented in Appendix S3).

Once the patches are identified and their size evaluated, various heavy tailed (e.g. power law, power law with a cut-off, log-normal, etc) and non-heavy tailed distributions (e.g. exponential) can be fitted to the patch-size distribution. One way of fitting a given distribution to data has been to use ordinary least square regression on the log-log transformed probability distribution function of patch sizes. However, this method is known to have substantial bias in estimating the parameter values, especially for small data sets [44], [45]. Maximum likelihood methods or least-square fits of the inverse-cumulative distribution (which quantifies the number of patches whose size is larger than a given value 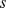 for different values of 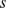) provide more accurate estimates of the parameters of most heavy-tailed functions [48].

#### Trends

The above methods inform us about whether the spatial indicators for a given spatial data set are significantly different from those of random patterns. However, to anticipate ecological transitions, we also need to know how these spatial indicators are changing along a degradation gradient. Trend statistics like the Kendall's 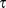 [12], [30] or Pearson's correlation coefficient [25], [49] can be used to quantify the strength of the trend in the indicator along the gradient.

### Simulated spatial data sets

Spatial data in ecology are typically obtained by field studies, data collecting devices placed at various locations of an ecosystem or extracted from spatial imagery. In any of these cases, the nature of data at a given spatial location can be of two types: (a) a discrete occupancy data, such as presence or absence of vegetation (or species) at each pixel of an image, or (b) a continuous variable, such as NDVI (Normalized Difference Vegetation Index) at each pixel or nutrient concentration at each sampling point.

Here, to serve better our method-illustration purposes, we chose to work on model-generated data rather than real data. We thereby circumvent limitations of missing or noisy data, and avoid issues of misinterpretation arising from potential insufficient knowledge about the underlying degradation gradients in real data. We generated three synthetic data sets using three representative models of tipping points and self-organization in ecological systems. The three models treat ecological variables in a spatially-explicit framework with stochasticity. They all describe vegetation dynamics under resource limitation or grazing pressure, but they differ in the nature of ecological interactions and the emerging spatial vegetation structure. A detailed description of the models can be found in Appendix S2 but a brief description follows.

**Figure 1:**
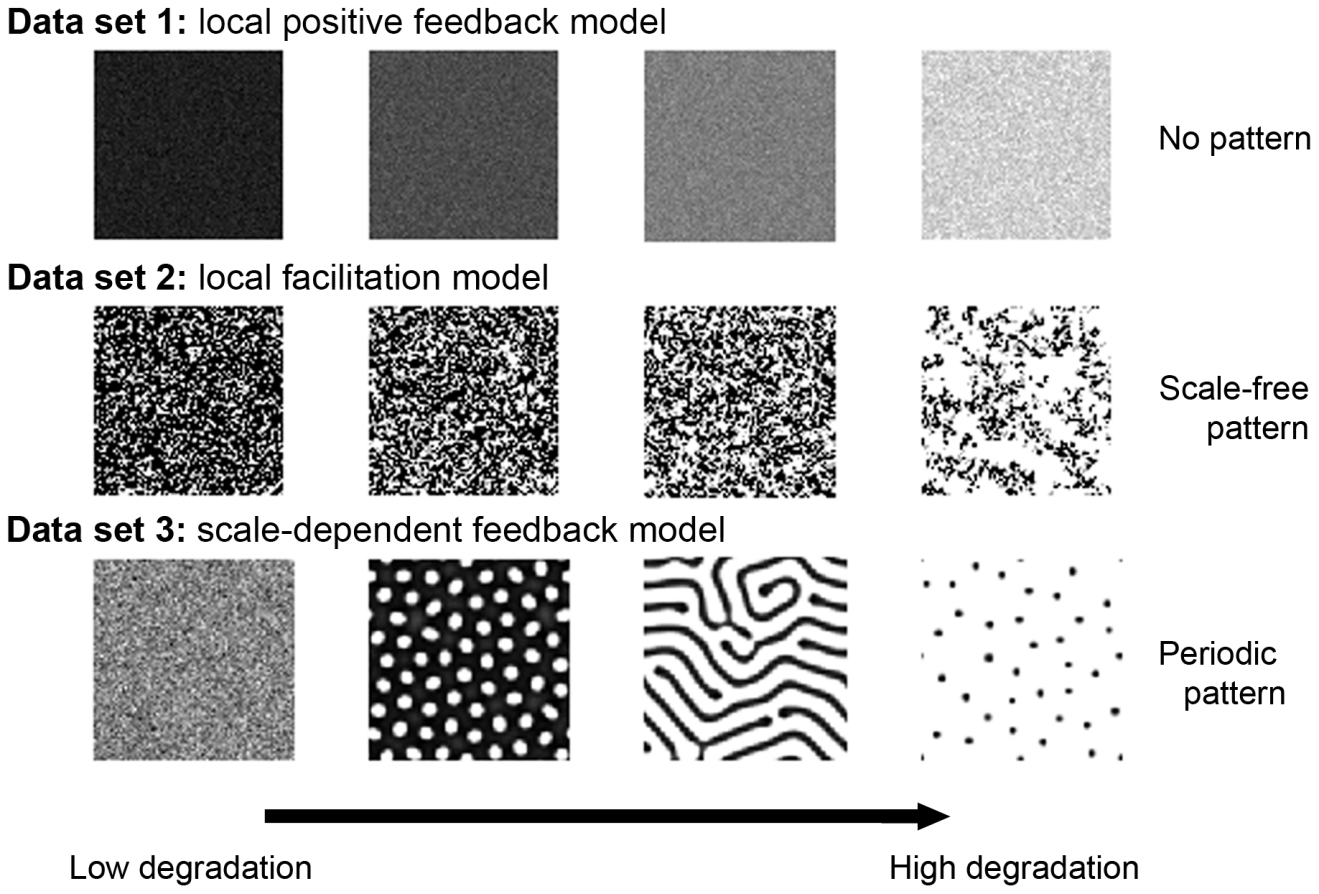
Spatial patterns along a degradation gradient in the three data sets. In each row, the system approaches the bifurcation point from left to right (see Fig. S1 in Appendix S2 to visualize the location of the four snapshots along the degradation gradient.) First row: local positive feedback model (data set 1). Middle row: local facilitation model (data set 2). Bottom row: scale-dependent feedback model (data set 3). In each panel, darker cells correspond to higher vegetation biomass.

- Data set 1 was obtained from a local positive feedback model resulting in a non-patchy vegetation structure [50], [51] (Fig. 1 first row). Space is represented as a two-dimensional lattice [52], [53]. Locally, vegetation density grows logistically and is lost due to grazing. Biomass and water are exchanged between neighboring sites at a certain rate, such that a site with high biomass (or water) will have the tendency to diffuse biomass (or water) to its neighboring sites. As rainfall falls below a certain threshold, the ecosystem undergoes an abrupt transition from a globally high vegetation density to a bare state due to the nearly synchronous shifts of each of the sites to a desert state [52].
- Data set 2 is based on a local facilitation model that exhibits spatial patterns characterized by a scale-free patch-size distribution [15]. In this stochastic cellular automaton model, an ecosystem is represented by a grid of cells, each of which can be in one of three discrete states: vegetated (+), empty (o) or degraded (−). Empty cells represent fertile soil whereas degraded cells represented eroded soil patches unsuitable for recolonization by vegetation. A key ecological mechanism is the positive effect of vegetation on its local neighborhood through increased regeneration of degraded cells. Because of this local facilitation, vegetated cells tend to form clusters (Fig. 1 second row). When the environmental conditions become harsher, there is a point at which the vegetation dies out and the system becomes a desert resembling a saddle-node (or fold) bifurcation.
- Data set 3 is based on a scale-dependent feedback model that results in periodic (Turing-like) spatial vegetation patterns [54]. This model is based on a three partial differential equations model describing the dynamics of vegetation biomass, soil water and surface water. Plants grow due to soil water availability and die due to natural mortality and/or grazing. The infiltration rate of water in the soil is higher in areas with vegetation than in bare soil, leading to the accumulation of water under vegetation and to its depletion further away, resulting in a scale-dependent feedback responsible for the formation of regular vegetation patterns [54] (Fig. 1 last row). When water availability becomes limited, a homogeneous vegetated state becomes unstable leading to self-organized patterns such as gaps, labyrinths and spots. A further reduction in water availability leads to a transition into a desert state, again mimicking a fold-like bifurcation.

The three models exhibit a bifurcation from one state (e.g., vegetated) to an alternative state (e.g., desert) as an external parameter (such as rainfall, grazing, etc) changes. See Appendix S2 for underlying mathematical equations and parameter values of these models. In a previous study [12], it has been shown that the three systems take increasingly longer to recover to their equilibrium after perturbation, thus demonstrating that critical slowing down is a generic feature of the transitions observed in these three ecological models, regardless of their different underlying mechanisms and their different types of spatial structures.

For our analysis, we selected ten snapshots (i.e. two-dimentional space discretized into matrices recording the spatial spread of the vegetation at the end of the simulation) for each of these models at different points along a gradient of degrading conditions prior to the transition. We illustrate our analyses using only ten of these points which are not equally spaced along the gradient (their location is shown on Fig. S1 in Appendix S2).

We are interested in quantifying how the spatial characteristics of these matrices change when approaching a tipping, or bifurcation, point. We are considering cases where the whole ecosystem shifts to an alternative state. In our mathematical representation of the ecosystem, this is equivalent to the entire matrix undergoing a shift. The degradation sequence of the matrices might correspond to snapshots in time (e.g. temperature changing through time) or in space (e.g. herbivory pressure changing in space depending on a distance to a water point). Both types of data are relevant to evaluate and test early warning signals. However, shifts of a given spatial ecosystem in time are more commonly the type of phenomena that we are trying to anticipate.

## Results

**Figure 2:**
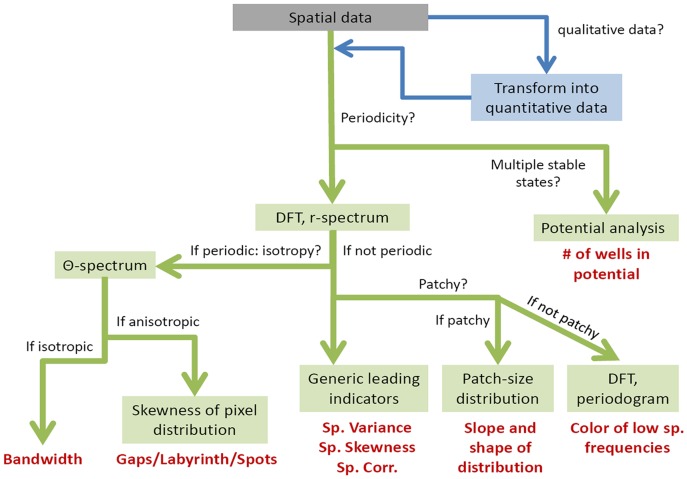
Flow chart of analysis to perform on a spatial data set.

We suggest a step by step process to decide which spatial indicators should be used (Fig. 2). The spatial statistics that need to be evaluated depend on the type of data set (with discrete or continuous values) at hand. Two of the three datasets used in this paper provide quantitative, continuous data, i.e. vegetation biomass (data set 1 and 3), whereas data set 2 gives qualitative, discrete data indicating the presence or absence of vegetation in each cell. For this latter data set, we transformed the original matrix by the coarse-graining procedure described in “Stastistical significance tests”. More specifically, we used submatrices of 5×5 cells in which we counted the number of cells occupied by vegetation [12]. We obtained matrices that were 25 times smaller than the original ones and where values in each of the cells ranged between 0 and 25, indicating the local abundance of vegetation. The size of the submatrix used to transform the original data may affect the behavior of the indicators.

The first question to ask is whether the patterns observed are periodic or not. The 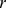-spectrum obtained from the DFT analysis provides information about whether the patterns are periodic, while the 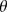-spectrum indicates whether the patterns are isotropic (i.e. with no specific orientation) or anisotropic (i.e. with a specific orientation, e.g. band-like patterns). If the patterns are not periodic, the generic leading indicators (i.e. spatial variance, spatial skewness and spatial correlation between nearby sites) may be used [12] and the power spectrum should be checked for possible reddening [13]. In addition, if the patterns are not only irregular but also patchy (e.g. can be characterized by two phases, one vegetated and one bare), the patch-size distribution may be plotted and estimated [15], [46]. If the patterns are periodic and anisotropic, the wavelength of the pattern should be evaluated. The wavelength is equivalent to the dominant length scale of the pattern provided by the 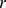-spectrum [43]. For periodic isotropic patterns, the skewness of the distribution of values of the data set (e.g. grey pixels in the case of a greyscale image) indicates the type of patterns (i.e. spots, gaps or labyrinths) [43]. Additionally, it is noteworthy that if the data set includes several replicates at each stress level, potential analysis may be performed [55]–[57] (see more details in Appendix S1).

Next, we present how this methodology can be applied to our three data sets.

### 1. Distinguishing periodic from non-periodic patterns

**Figure 3:**
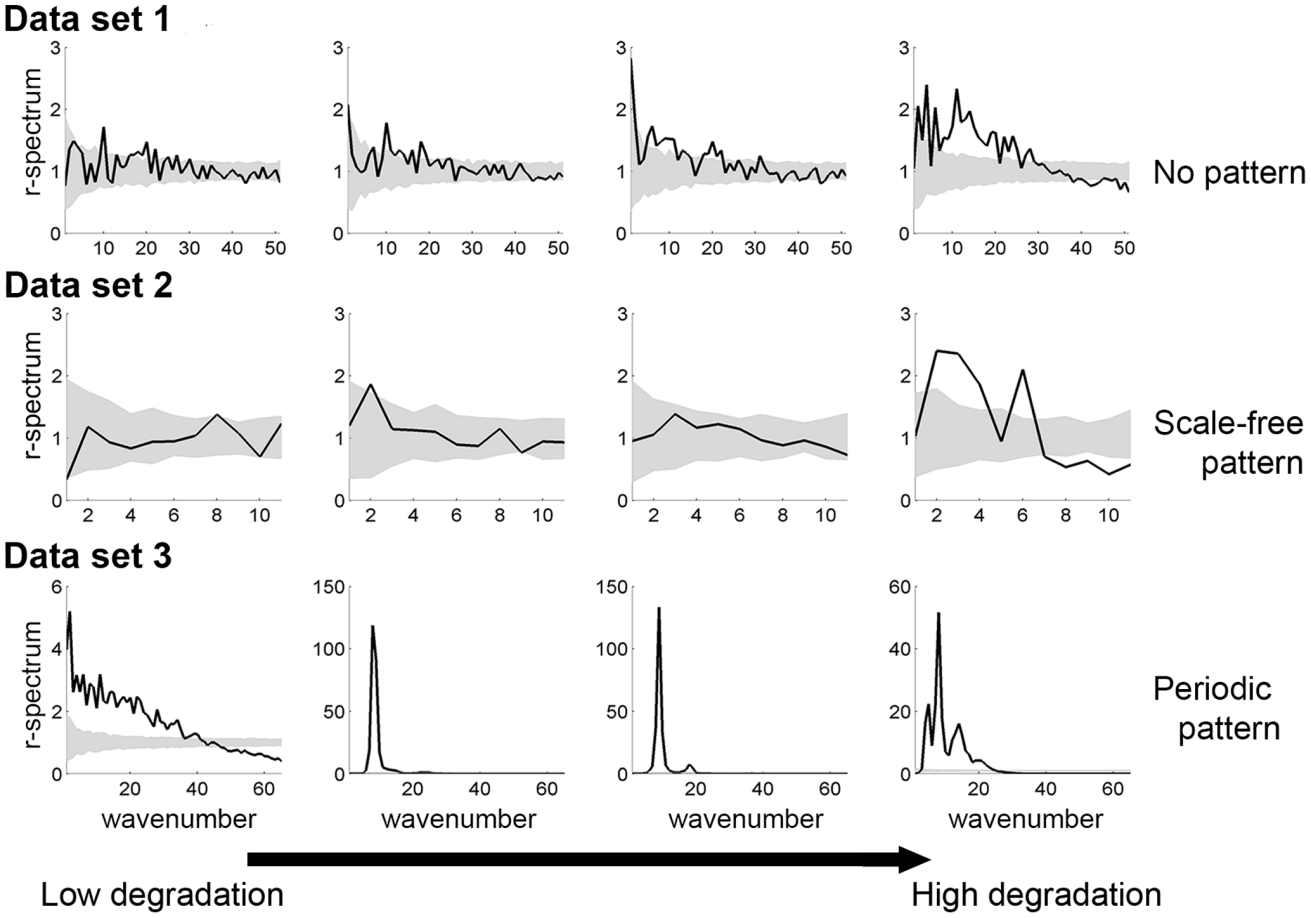
Radial-spectrum along a degradation gradient in the three data sets. In a row, each panel corresponds to the radial-spectrum of the system at a different location along the degradation gradient. The system approaches the bifurcation point from left to right column (as in Fig. 1). First row: local positive feedback model. Middle row: local facilitation model (original data transformed using 5×5 submatrices). Bottom row: scale-dependent feedback model. Gray areas correspond to 95% confidence intervals obtained using 200 simulations of a null model (i.e. data sets of same size generated by reshuffling the original data set).

We used DFT analysis to estimate the 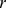-spectra as a function of wavenumbers for all the three data sets (Fig. 3). The first and second data sets (Fig. 3, first and second row) show a noisy pattern indicating that contribution to 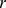-spectra is not significant for all wavenumbers. However, the 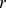-spectrum for the third data set, the scale-dependent feedback model, shows a clear peak (Fig. 3 last row) even far from the transition. The peak indicates that there is dominant wavelength (corresponding to a characteristic patch size) which is a signature of periodic patterns. Note that periodicity can also be seen by plotting the power spectrum, where the periodicity is visible as a ring corresponding to the dominant wavelength of the data set (see upcoming paragraph “DFT and reddening”). Therefore, we conclude that the two first data sets are not periodic whereas the last one shows some spatial periodicity.

### 2. Probing spatial early warnings for non-periodic patterns

We first focus on the case of the non-periodic patterns, i.e. data set 1 showing no clear spatial structure and data set 2 with vegetation clusters.

#### Spatial correlation, variance and skewness

**Figure 4:**
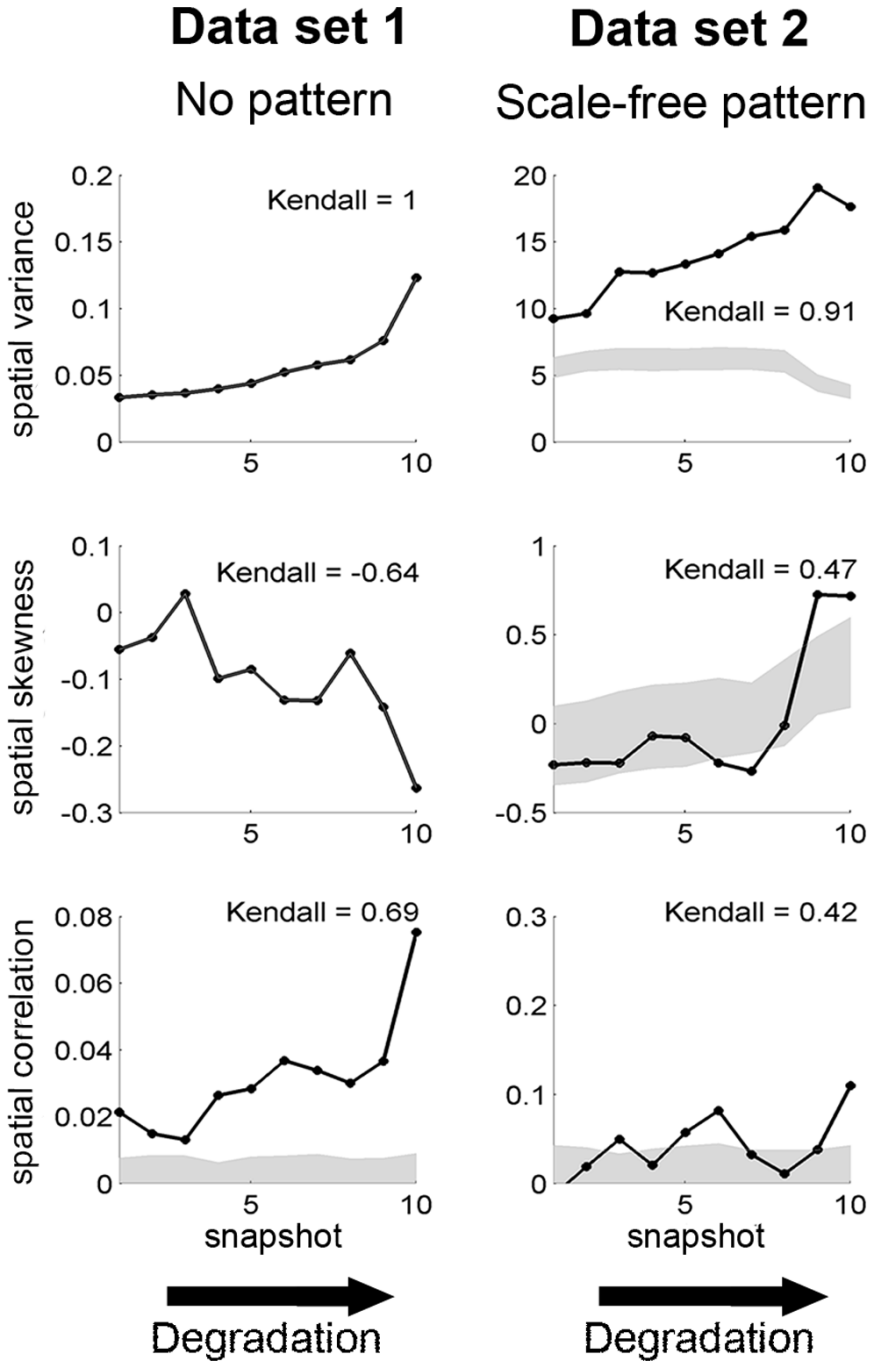
Generic leading indicators in data sets 1 and 2 along a degradation gradient. In each panel, the 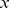-values correspond to the rank of the snapshot of the system along the degradation gradient. This mimicks a scenario where we would not know the exact value of the driver but where we can order the data set along a degradation gradient and see Fig. S1 in Appendix S2 to visualize the location of the four snapshots along the degradation gradient. For each of the three data sets, 10 snapshots were used. Left: local positive feedback model (data set 1). Right: local facilitation model (data set 2; original data transformed using 5×5 sub-matrices). First row: spatial variance. Second row: spatial skewness. Third row: spatial correlation at lag one. In each panel, Kendall's 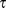, quantifying the trend of the indicator, is indicated. Gray areas correspond to 95% confidence intervals obtained using 200 simulations of a null model (i.e. data sets of same size generated by reshuffling the original data set).

In data set 1, spatial correlation at lag 1 and spatial variance increase whereas spatial skewness decreases toward the bifurcation point (Fig. 4), as expected from theory. The behavior of these indicators is very similar for data set 2, except that the spatial variance decreases just before the collapse. This difference in the behaviour of the spatial variance is due to the fact that data set 2 measures only presence or absence of vegetation (i.e qualitative data) whereas data set 1 provides biomass density at each location in space (i.e. quantitative information). We note that the spatial variance and skewness for data set 1 are identical to those of null model which was obtained by a random reshuffling; therefore, we do not see error bars. However, we used the coarse-graining method for the discrete data set 2 which provides a null model for spatial variance and skewness. See a later section on ‘Probing statistical significance’ for further comments.

#### DFT and reddening

**Figure 5:**
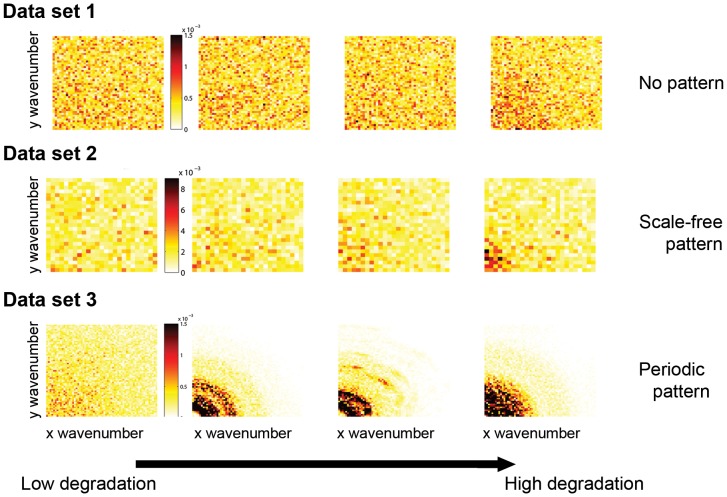
Power spectrum along a degradation gradient in the three data sets. In a row, the system approaches the bifurcation point, from left to right column (as in Fig. 1). For a data set of size 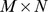, the power spectrum is typically plotted for wavenumbers up to 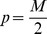 and 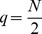 [47] and is scaled by the spatial variance 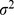 (i.e. the scaled power spectrum is evaluated as 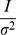) [35]. Red color indicates higher values of the scaled power spectrum, 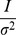. The 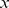 and 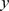-axis correspond to the wavenumbers along these directions. First row: local positive feedback model (data set 1). Middle row: local facilitation model (data set 2; original data transformed using 5×5 sub-matrices). Bottom row: scale-dependent feedback model (data set 3).

The spatial power spectrum (or 2D-periodogram, see Eq. 5 in Appendix S1) shows a reddening of the signal, i.e., the amplitude of the power spectra increases at low wavenumbers, as the system approaches the bifurcation point (Fig. 5 first and second rows). The reddening of the power spectra provides advance warning of the transition in all data sets. That trend is even clearer on the 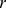-spectra, which sums the values of the 2D-periodogram for all the wavenumbers and shows that the lower wavenumbers contribute more to the total variance of the data set as the system approaches the bifurcation point (Fig. 3 first and second rows).

#### Non-periodic and patchy: patch-size distribution

**Figure 6:**
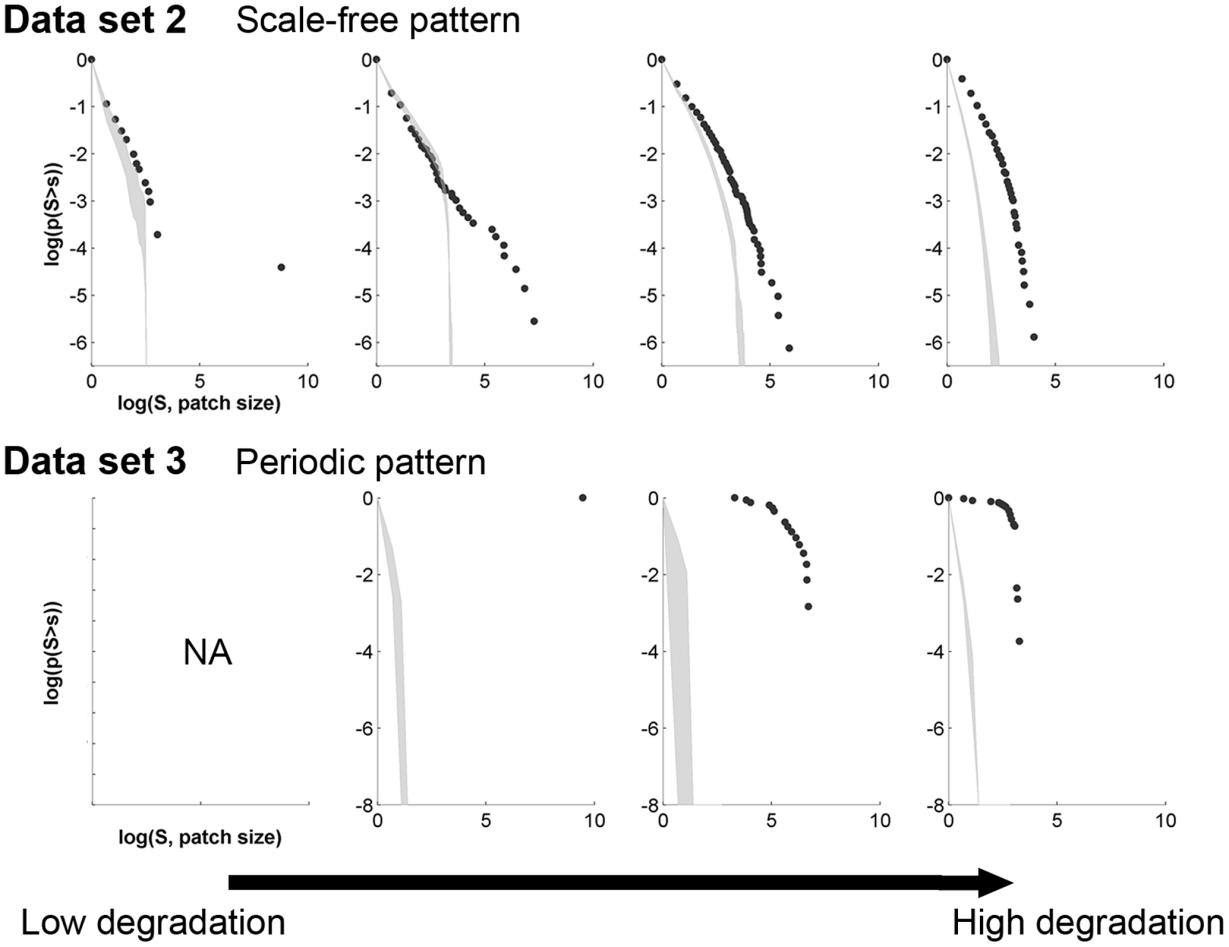
Inverse cumulative patch-size distributions along a degradation gradient in data sets 2 and 3. Along each row, the system approaches the bifurcation point from left to right colum (as in Fig. 1)n. First row: local facilitation model (original data not transformed). Bottom row: scale-dependent feedback model. Gray areas corresponds to 95% confidence interval obtained using 200 simulations of a null model (i.e. data sets of same size generated by reshuffling the original matrix).

Data set 2 was not only characterized by non-periodic patterns, but our visual examination reveals that it also exhibits distinct patches of vegetation and bare ground. In that case, it makes sense to look at the distribution of patch sizes. A way of plotting such data is to calculate the inverse cumulative distribution, i.e. plotting the number of patches whose size is larger than a given value 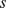 as a function of 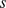. The inverse cumulative distribution is nearly scale-free far from the bifurcation point, while its slope decreases and the distribution becomes bent (toward less large patches) as the system approaches the bifurcation point (Fig. 6 top row) [46].

For comparison, we plotted the inverse cumulative patch-size distribution of data set 3 that is also patchy but periodic. Far from the transition, after the onset of pattern formation, the periodic patterns presented a patch-size distribution characterized by a sharp cutoff (Fig. 6 bottom row). As the system approaches the bifurcation point, the value of the cutoff decreases indicating decreasing patch size in the periodic pattern.

### 3. Probing spatial early warnings for periodic patterns

**Figure 7:**
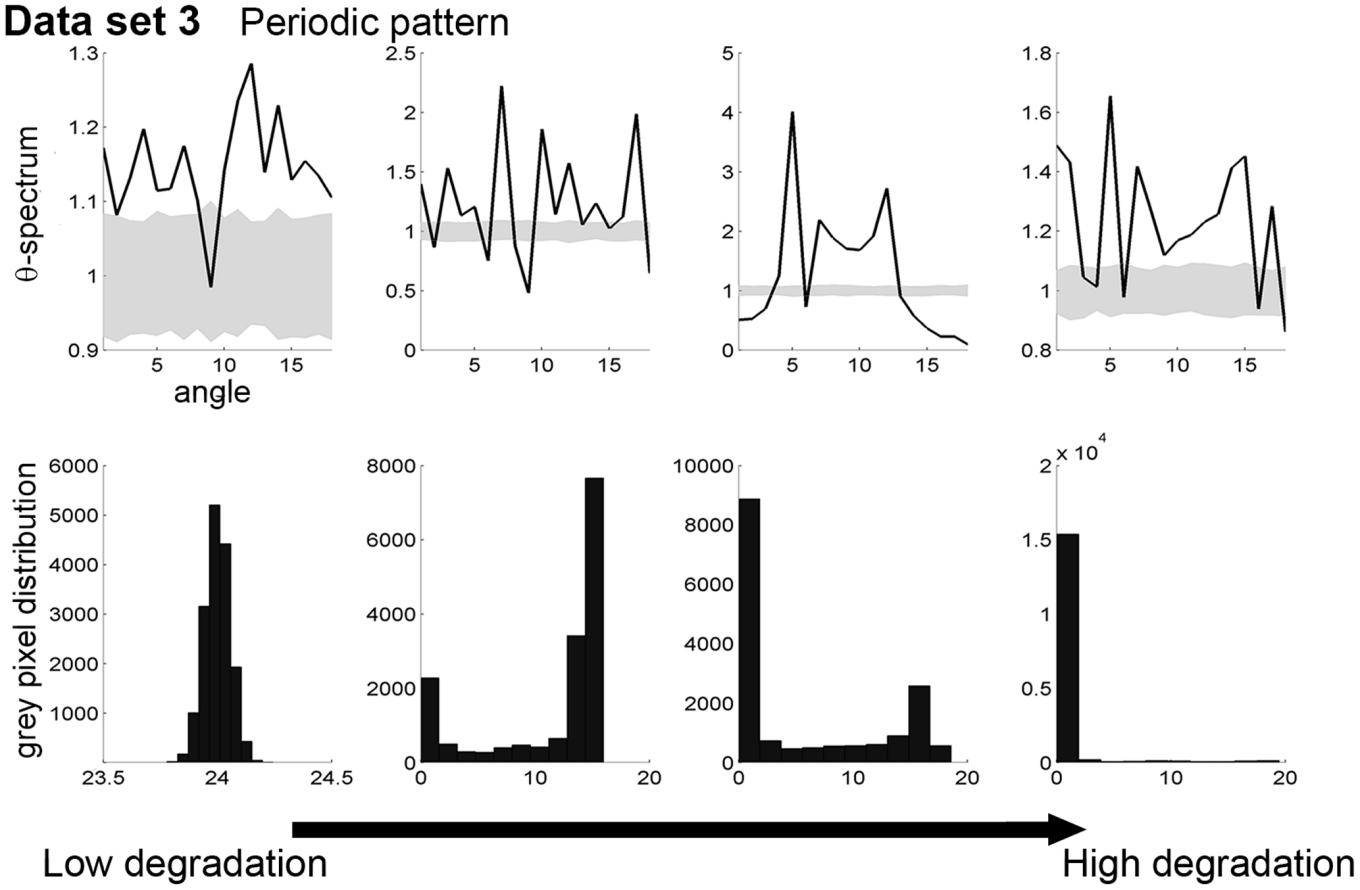
Analysis of the periodic patterns of data set 3. In a row, the system approaches the bifurcation point from left to right column (as in Fig. 1). First row: 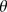-spectrum. Gray areas corresponds to 95% confidence interval obtained using 200 simulations of a null model (i.e. data sets of same size generated by reshuffling the original matrix). Second row: histogram of the values of the data set (or pixels of the image).

In contrast to the first two data sets, data set 3 exhibits periodic patterns (Fig. 1 and 3 last rows). The 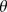-spectra does not indicate a strong and clear signal at any specific angle, suggesting that the patterns do not have a clear orientation, i.e. patterns are isotropic (Fig. 7 first row). When the system approaches the bifurcation point (from left to right panel on Fig. 7 second row), the distribution of values of the data set goes from one peak reflecting the absence of patterns, to a two-peak distribution due to the occurrence of both vegetation and bare soil in the system after the emergence of spatial patterns, and finally to a distribution that is skewed toward small values because of the dominance of bare soil in the system. The last distribution observed before the bifurcation point characterizes spot patterns [43] which has been hypothesized to be an indicator of imminent desertification [22].

### 4. Probing statistical significance

**Figure 8:**
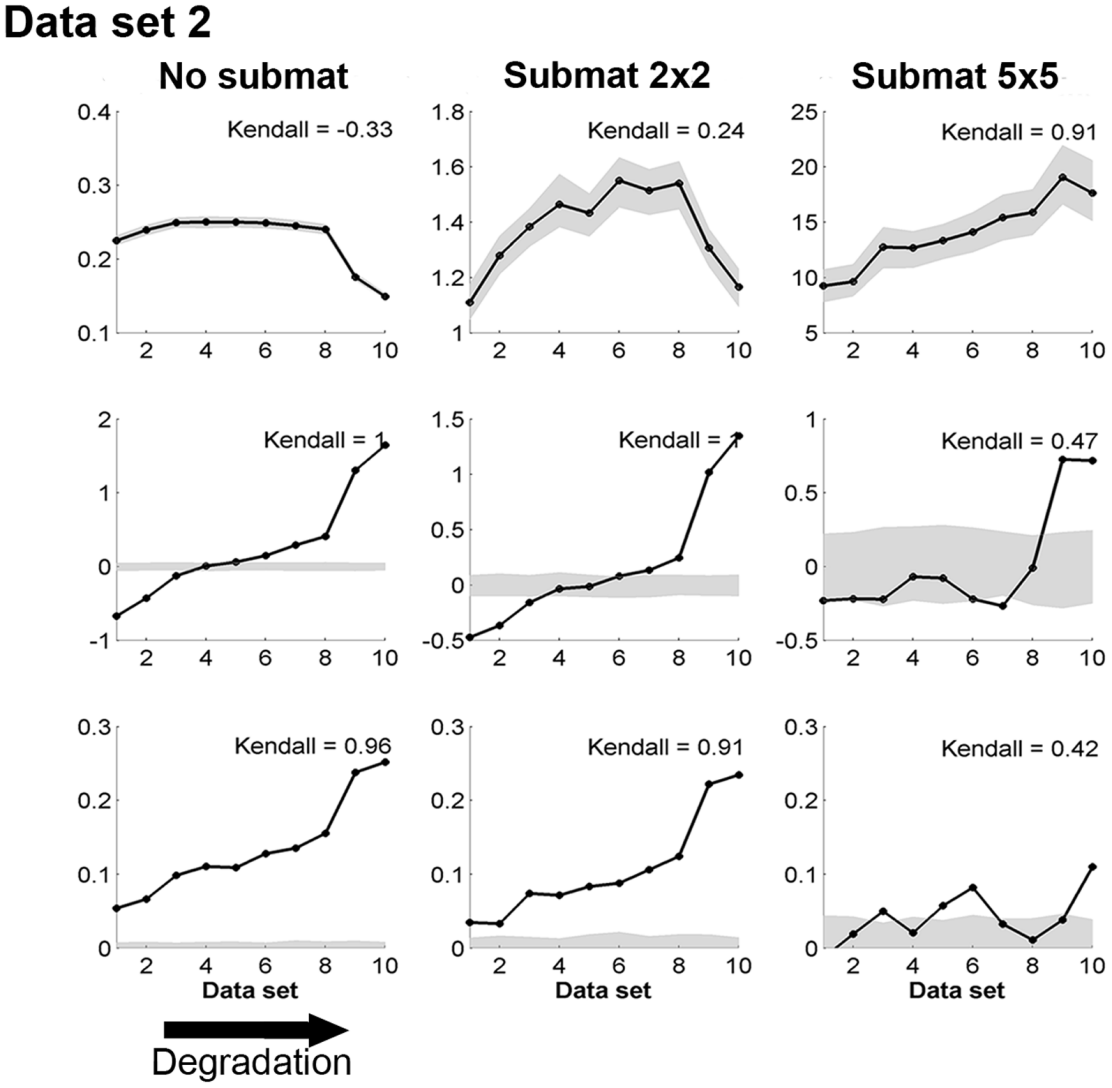
Generic leading indicators in data set 2 along a degradation gradient using different coarsening of the original data. In each panel, the 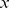-value indicates the rank of the snapshot along the degradation gradient. This mimicks a scenario where we would not know the exact value of the driver but where we can order the data set along a gradient and See Fig. S1 in Appendix S2 to visualize the location of the four snapshots along the degradation gradient. Left: original data. Middle: original data transformed using 2×2 sub-matrices. Middle: original data transformed using 5×5 sub-matrices. First row: spatial variance. Second row: spatial skewness. Third row: spatial correlation at lag one. In each panel, Kendall's 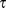 quantifies the strength of the trend of the indicator. Gray areas correspond to 95% confidence intervals obtained using 200 simulations of a null model (i.e. data sets of same size generated by reshuffling the original matrix).

We make further comments on the statistical significance for indicators of spatial data, especially in the context of null models for spatial variance and spatial skewness. As an illustration of the coarse-graining method proposed in the “Methods” section, we compared the trends in the generic leading indicators on coarse-grained matrix obtained by different dimensions of submatrix, 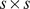 with 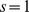 (in this case the coarse-grained matrix is identical to the original matrix), 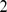 and 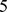 but starting from the same original matrix of size 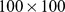 from our data set 2. As shown in the first row of Fig. 8, the values of spatial variance along the degradation gradient differ substantially from the ones of the null model only in the coarse-grained case (second and third column). The same result seems to be true, although the differences are not as pronounced, for spatial skewness (second row). In contrast, the coarse-graining method does not offer a good null model for spatial correlation (last row, Fig. 8). In summary, a random matrix that has the same dimensions and average as the original spatial data can act as a null model for computing spatial correlation only. On the other hand, a comparison between indicators of coarse-grained matrices of both original and random matrix data can act as a null model for spatial variance and spatial skewness.

## Discussion

In this manuscript, we presented a systematic methodology for applying spatial early warning signals of abrupt ecological transitions. To demonstrate the methods, we employed data generated from simulations of spatially-explicit ecological models with stochasticity that showed abrupt transitions from one state to an alternative state. It is increasingly being recognized that spatial dynamics pose challenges and provide opportunities for both basic science as well as management [58]. Research on spatial dynamics in ecology is continually uncovering new patterns and mechanisms. Some of these processes are likely related to regime shifts. In this context, the main objective of this manuscript was to provide a methodological guide that can stimulate the application of spatial indicators for ecological transitions on empirical data sets from real case studies.

In recent years, much effort has been devoted to the search for ‘generic’ indicators based on the idea that there are some common behaviors across a range of complex systems as they approach a bifurcation point [59]. Taking into account the spatial organization of natural systems has revealed that many indicators may not behave in spatially-structured systems as they would in other systems. More specifically, recent theoretical studies have suggested that trends of generic leading indicators (spatial variance, spatial skewness, and spatial correlation between nearby sites) could be different in self organized patterned systems [12]. For such ecosystems, system-specific indicators may be more appropriate. In particular, when spatial patterns are periodic or regular (Turing-like), the shape of the patterns may give an idea of the proximity to the threshold where the system may undergo a regime shift [22]; specifically, spots could warn of approaching desertification [16], [22]. If the patterns are non-periodic or irregular (in particular, if the patch-size distribution is described by a heavy-tailed distribution), the patch size distribution may contain information about the degradation level of the ecosystem but more needs to be known about the underlying ecological mechanisms to interpret the changes in the shape of this distribution [15], [20], [46], [60]. In other words, the nature of spatial organization of ecosystems seems to be a key factor in determining what type of indicators may be employed to detect an impending ecological transition in spatially-structured systems. Therefore, a first and essential step when starting to analyze a spatial data set is to get an idea of the type of spatial organization that one is dealing with. A good knowledge of the system and its underlying ecological mechanisms (specifically those responsible for the spatial structure) are required to know which indicator to use and how to interpret the changes. Theoretical studies have started developing methodologies for inferring underlying mechanisms from a limited number of spatial snapshots [61], [62]. Such knowledge will facilitate to assess the risks of ecological transitions while accounting for potential false and failed alarms.

Clearly, the usefulness of the indicators presented here depends greatly on the underlying mechanisms driving the change in the ecosystems studied. In all our analyses, we assumed that a single driver is monotonically changing, while the underlying environmental conditions are assumed relatively stable and the environmental stochasticity relatively weak. This may not always be the case. For example, spatial correlation can increase due to changes in the underlying spatial heterogeneity of the environment, or due to alterations in local ‘mixing’ (or diffusion) levels in the landscape [5]. Effects of increasing spatial variance near a critical point could be confounded by various intrinsic factors such as demographic noise arising from changes in population sizes, state-dependent/multiplicative noise. The nature of the dispersal processes between patches in fragmented landscapes may affect expected trends in the indicators. In addition, extrinsic factors such as environmental fluctuations that vary in space and time can complicate our interpretations of early warning signals. In a similar way, system-specific conditions may affect the behavior and thereby interpretation of the indicators. For example, in Mediterranean drylands, degradation is accompanied by a change in the patch-size distribution toward less large patches, whereas the opposite was observed in salt marshes because the underlying mechanisms driving the formation of the patterns differ in the two ecosystems [15], [46], [60]. Therefore, our interpretations of leading indicators are prone to both false positives and false negatives.

To prevent such errors, knowledge of the underlying heterogeneity, repeated observations of the system [60], or a sufficiently well-described system so that it can be modelled are necessary to know what to expect along a degradation trend. As this is not usually the case, general models have been suggested to be fitted to time series data[63], [64] to simulate surrogate scenarios for comparing trends obtained from the original data sets. In this case the model provides an expectation of i) whether and when a shift is likely, and ii) what trends should look like as the system is approaching a shift. Such approaches, however, are yet to be developed for spatially-explicit systems. Although we presented a couple of ways to develop null models, these are rather simplistic as they entirely neglect any underlying spatial structuring. Therefore, the design and selection of null models for spatial data, which is an area of active research in ecology, is another important avenue for further research [65]. There are other promising avenues of research to be pursued. Composite metrics that combine spatial patterns with their temporal dynamics could potentially offer new and potentially more reliable indicators of imminent transitions [66]. Ecological transitions and leading indicators in the context of metapopulation dynamics, where factors that stabilize or make species more vulnerable to extinction have been extensively studied, may be a fertile ground for further research.

More importantly, despite a few recent studies, we still lack empirical tests of spatial early warnings. Researchers have studied spatial warnings in laboratory populations of microbial organisms such as Daphnia and yeast [25], [31]. In these studies, individual populations are maintained in locally well-mixed small beakers or petri-dishes and they are ‘connected’ to other populations by ‘controlled dispersal’ where the researcher transfers a fraction of the local population to its nearest neighbors. In the field, researchers have employed space-for-time substitution; in this approach, it is assumed that spatial patterns at locations with different values of stressors (e.g. grazing or rainfall) are equivalent to the dynamics of spatial patterns where the stressor is changing with time. This is a widely used approach in ecology as an alternative to long term ecological studies, for example to investigate ecological succession or how ecosystems may respond to climate change [67]. In the context of alternative stable states and tipping points, this method has been employed to establish the existence of (multiple) stable states in savanna ecosystems as a function of rainfall [57], [68], and to forecast how spatial self-organization of semi-arid vegetation may respond to increasing stressors such as grazing [15]. However, the application of space-for-time method is not without limitations [69]. One needs to be cautious about the possibility of existence of various other sources of heterogeneity, both biotic and abiotic, along a spatial gradient of stressor. As we have argued before, the interpretation of the trends of early warnings are crucially dependent on the nature of local ecological interactions and the patterns they produce. Therefore, sources of heterogeneity, especially those that alter the ecological processes generating spatial patterns, may therefore compound the complexity of interpretation of results based on space-for-time substitutions.

In conclusion, both the theory and the application of the spatial indicators lag behind the development of the temporal ones. Spatial patterns may however offer advantages for anticipating or detecting ecological transitions of the types studied here [11], [32]. Unlike temporal indicators which require long, unbroken time series of frequent observations, spatial indicators may be evaluated even if measurements are irregular or infrequent over time. While spatial pattern measurements require intensive data collection at each time point, in many cases this may be easier than high-frequency time series sampling [5], [11], [13]. In both space and time, more empirical validation of the indicators currently proposed in the literature is needed. We do not yet have an example where early warning signals were used to avert an upcoming shift (they have been used in models, experiments or retroactively). We hope that this work will stimulate further development, testing and application of spatial indicators in a broad range of ecosystems.

## Supporting Information

Appendix S1 **Spatial indicators.**
(PDF)

Appendix S2 **The three models used to generate the test data sets.**
(PDF)

Appendix S3 **Results compared to the null model based on white noise.**
(PDF)
