# Supplementary material for: Early Warning Signals of Ecological Transitions: Methods for Spatial Patterns
Source: PLoS One. 2014 Mar 21;9(3):e92097. doi: 10.1371/journal.pone.0092097 (PMC3962379; doi:10.1371/journal.pone.0092097)
Supplement: Appendix S1 — Spatial indicators. (PDF) [file pone.0092097.s001.pdf]

Early warning signals of ecological transitions: Methods for spatial patterns

Sonia Kéfi[1,‡*], Vishwesha Guttal[2,‡], William A. Brock[3,4], Stephen R. Carpenter[4,5], Aaron M. Ellison[6], Valerie N. Livina[7], David A. Seekell[8], Marten Scheffer[9], Egbert H. van Nes[9], Vasilis Dakos[10]

[1] Institut des Sciences de l'Evolution, CNRS, Université de Montpellier II, Montpellier, France

[2] Centre for Ecological Sciences, Indian Institute of Science, Bangalore, India

[3] Department of Economics, University of Wisconsin, Madison, Wisconsin, United States of America

[4] Department of Economics, University of Missouri, Columbia, Missouri

[5] Center for Limnology, University of Wisconsin, Madison, Wisconsin, United States of America

[6] Harvard Forest, Harvard University, Petersham, Massachusetts, United States of America

[7] National Physical Laboratory, Hampton Road, Teddington TW11 0LW, United Kingdom

[8] Department of Environmental Sciences, University of Virginia, Charlottesville, Virginia, United States of America

[9] Department of Aquatic Ecology and Water Quality Management, Wageningen University, Wageningen, The Netherlands

[10] Integrative Ecology Group, Estacion Biologica de Donana, Sevilla, Spain

‡ These authors contributed equally to this work

∗ To whom correspondence should be addressed: sonia.kefi@univ-montp2.fr

Appendix S1: Spatial indicators

A spatially-explicit representation of an ecosystem can be an array of locally-coupled units in which an ecologically relevant quantity is estimated, such as biomass or nutrients. In the rest of the paper, we assume that our data sets are composed of snapshots, each of which is a two-dimensional space discretized into M and N units in x and y directions, respectively. Therefore, we have a total of $M \times N$ units of equal size for each snapshot. We call $z[m, n]$ the value of the local state variable at location $\mathbf{x} = (m, n)$, where m and n can take values $1, 2, ..., M$, and $1, 2, ..., N$, respectively. We are interested in quantifying how the spatial characteristics of this matrix change when approaching a tipping, or bifurcation, point.

We are considering cases where the whole ecosystem shifts to an alternative state. In our mathematical representation of the ecosystem, this is equivalent to the entire matrix undergoing a shift. The degradation sequence of the matrices might correspond to snapshots in time (e.g. temperature changing through time) or in space (e.g. herbivory pressure changing in space depending on a distance to a water point). Both types of data are relevant to evaluate and test early warning signals. However, shifts of a given spatial ecosystem in time are more commonly the type of phenomena that we are trying to anticipate.

Indicators based on slowing down

The first set of indicators are direct consequence of the fact that as a system approaches a bifurcation point, it takes increasingly long to return to its local equilibrium after a perturbation. This slowing down affects the spatial dynamics of the ecological system [1] and leads to specific spatial signatures prior to the transition point.

Spatial correlation

Due to increased recovery time to local equilibrium, when a system approaches a bifurcation point, spatial coupling, or flow, between neighboring units becomes the dominant force. This favors neighboring units to become more like each other, i.e. they become increasingly correlated [2]. The increasing spatial coherence can be quantified by the spatial correlation function, or Moran's I, between ecological states $z[i,j]$ and $z[m,n]$ separated by a distance r:

$$C_2(r) = \frac{MN\sum_{i=1}^{M}\sum_{m=1}^{M}\sum_{j=1}^{N}\sum_{n=1}^{N} w[i,j;m,n](z[i,j]-\bar{z})(z[m,n]-\bar{z})}{W\sum_{m=1}^{M}\sum_{n=1}^{N}(z[m,n]-\bar{z})^2} \tag{1}$$

where $\bar{z}$ is the spatial mean of the state variable ($\bar{z} = \sum_{m=1}^{M}\sum_{n=1}^{N} z[m,n]/(MN)$), $w[i,j;m,n]$ is 1 if spatial units $[i,j]$ and $[m,n]$ are separated by a distance r, and is 0 otherwise, W is the total number of units separated by the distance r. In the notation $C_2(r)$, the subscript 2 stands for 2-dimensional space. The near-neighbor spatial correlation $C_2(\delta)$, the analog of autocorrelation at lag 1 for time series, is calculated for the distance δ corresponding nearest neighboring units of the system.

As the system approaches the bifurcation point, the spatial correlation function increases at all dis-

tances [2]. The correlation function itself, however, decays with distance meaning that state variables separated by farther distances are less correlated than those separated by shorter distances. Based on the correlation function, we can define a correlation length (denoted by ξ) as a measure of how fast the correlation decays with distance, or equivalently, as the spatial scale at which two spatial units become uncorrelated. This can be computed by e.g. fitting an exponential function $\exp(-r/\xi)$ to $C_2(r)$. The correlation length ξ thus obtained also increases as the system approaches a bifurcation point. In a recent empirical paper on population dynamics in a spatially-connected laboratory populations [3], Dai et al. proposed a related measure called 'recovery length' which is the spatial length scale at which a system recovers from a perturbation. For simplicity and ease of computation, here we have restricted ourselves to near-neighbor spatial correlation, $C_2(\delta)$, alone. We also note that the choice of an exponential fit is based on analogous changes between states in in physical materials known as 'phase transitions' where it is shown that exponential is a good fit for systems far from the point of phase transition. However, these studies also show that power-laws with negative exponents may be more suitable at the point of phase transition. In our study, we are approaching a critical point of abrupt ecological transition but are never exactly at the critical point. Therefore, we restrict ourselves to exponential fits alone (but see 'Patch-based indicators' below).

Spatial spectral properties (DFT)

Let us recall the definition of spectral properties in the context of time series before drawing parallels with the spatial scenario. Time series data can be decomposed into components of different sine and cosine waves of different frequencies, where frequency is an inverse measure of the time period of the wave; a high frequency wave corresponds to small period whereas a low frequency corresponds to high period. The spectral density function computed as a function of frequency quantifies the relative contributions/weights of different frequencies of sine (or cosine) waves to the time series under study. A spatial data can similarly be quantified by strengths of different sine and cosine waves of different wavenumbers. A wavenumber can be thought of as a 'spatial frequency', or the number of times that a pattern is repeated in a unit of spatial length. When we think of spatial data, periodicity is visualized as wavelength, which is inversely related to wavenumber (i.e., a small wavenumber corresponds to a large wavelength and conversely). A spatial spectral density function is typically plotted as a function of wavenumbers and it quantifies the relative contributions/weights of different wavenumbers of sine (or cosine) waves to

the spatial data under study.

How does increased memory affect spectral properties in the spatial data? Increased memory manifests itself as spectral reddening, i.e. spatial variation will typically become increasingly concentrated at lower wavenumbers. Since wavenumbers and wavelengths are inversely related to each other, the long wavelength fluctuations become dominant prior to a transition [4]. Spatial spectral reddening is formally captured by the Discrete Fourier Transform (DFT) that decomposes spatial variation into a summation of periodic sine and cosine functions [5].

The Discrete Fourier Transform (DFT), $\hat{z}$, of a spatial state variable z is defined as:

$$\hat{z}[p,q] = \frac{1}{MN}\sum_{m=0}^{M-1}\sum_{n=0}^{N-1}(z[m,n]-\bar{z})e^{-i\,2\pi(mp/M+nq/N)} \quad (2)$$

$$= \frac{1}{MN}\sum_{m=0}^{M-1}\sum_{n=0}^{N-1}(z[m,n]-\bar{z})\Big(\cos\big(2\pi(\frac{mp}{M}+\frac{nq}{N})\big)-i\sin\big(2\pi(\frac{mp}{M}+\frac{nq}{N})\big)\Big) \quad (3)$$

$$= a[p,q]-i\,b[p,q] \quad (4)$$

where i is the imaginary number defined such that $i^2=-1$, $a[p,q]$ and $b[p,q]$ are the real and imaginary parts of DFT $\hat{z}[p,q]$, and symbols p and q correspond to wavenumbers in x- and y-dimensions (see equations 1 and 2 in [6]). Note that DFT, in general, can be a complex number, and therefore we plot the power spectrum $I[p,q]=|\hat{z}[p,q]|$, where $|\hat{z}|$ is the modulus of the complex number $\hat{z}$. The Fourier coefficients, $a[p,q]$ and $b[p,q]$, allow plotting the power spectrum I (also called 2D-periodogram) as:

$$I[p,q]=MN(a[p,q]^2+b[p,q]^2). \quad (5)$$

The power spectrum, $I[p,q]$, measures the contribution of cosine and sine waves with wavenumbers $[p,q]$ in the x- and y-coordinates, respectively, to the variance of the spatial data. The power spectrum is typically plotted for wavenumbers up to $p=\frac{M}{2}$ and $q=\frac{N}{2}$ [7] and is scaled by the spatial variance σ^2 (i.e. the scaled power spectrum is evaluated as $\frac{I}{\sigma^2}$) [6].

Finally, we remark that the spatial correlation function and spectral properties can be formally shown

to be related to one another under certain conditions (referred to as the Weiner-Kinchin theorem). Therefore, an increase in correlation length as computed from the spatial correlation function and spectral reddening i.e., increased contribution of small wavenumbers or large wavelength to the spatial fluctuations are equivalent quantification of the same underlying dynamics. However, depending on the nature of the dataset, spatial resolution, etc, one quantification may appear more informative than the other.

Variability-based indicators

Spatial variance and spatial skewness

Increased recovery time enroute to a bifurcation point may lead to stronger fluctuations around the equilibrium state of the system [8]. This can cause spatial variance of the system to increase prior to a transition [9, 10]. Spatial variance is formally defined as the second moment around the spatial mean of the state variable:

$$\sigma^2 = \frac{1}{MN} \sum_{n=1}^{N} \sum_{m=1}^{M} (z[m,n] - \bar{z})^2 \tag{6}$$

It has also been shown that the fluctuations around the mean can become increasingly asymmetric as the system approaches a bifurcation point. This is because the fluctuations in the direction of the alternative stable state take longer to return back to the equilibrium than those in the opposite direction [10]; this asymmetry can also arise due to local flickering events (i.e. occasional jumps of local units between their current and alternative state) [11]. This spatial asymmetry can be measured by spatial skewness, which is the third central moment scaled by the standard deviation:

$$\gamma = \frac{1}{MN} \sum_{n=1}^{N} \sum_{m=1}^{M} \frac{(z[m,n] - \bar{z})^3}{\sigma^3} \tag{7}$$

Potential analysis

For time series data, a statistical method referred to as potential analysis has been developed to detect whether a system exhibits alternative states [12, 13]. The underlying idea is that if a system has alternative states, it may sample these states when perturbed by sufficiently strong noise [14] that would result in a bimodal probability distribution of states when monitored in time [12, 13]. Potential analysis therefore identifies flickering [11, 15] and may warn of the existence of alternative states. However, it also

implies that the system is already undergoing shifts between its alternative states. In that sense, it is not an early warning of an approaching transition, but it rather helps detecting a bifurcation that is in progress. Potential analysis has been employed to detect tipping points in climate [12, 13, 16, 17].

In the same way as a system exhibiting alternative states may flicker in time between these states, a system found at different states at several locations where the environmental conditions are similar may be seen in its alternative states in space (analogous to flickering, but in space). Recently, this method was used to detect the existence of alternative states in spatial data of Savanna ecosystems [18, 19]. The application of this method to spatial data is only at its beginning but could provide interesting insights into identifying the co-existence of alternative states in space, and thus warn about the possibility of regime shifts of whole ecosystems in time.

The method assumes that the data may be approximated by a stochastic potential equation of the type [13]

$$dz = -U'(z)dt + \sigma dW \tag{8}$$

where z is the dynamical state variable, σ is the strength of the external fluctuations and dW represents a stochastic term called the Wiener process.

The probability density of the data is then used to reconstruct the potential of the system [12], where the potential is a function whose minima (respectively maxima) indicate the stable (respectively unstable) ecological states of the system:

$$U \sim -\frac{\sigma^2}{2} x \log(P(z)) \tag{9}$$

with U the potential function, σ the strength of the stochastic fluctuations, and $P(z)$ the probability density function obtained from the data.

In practice, the probability density of the system is fitted with polynomials of increasing orders until a negative leading coefficient is encountered; details of the method are presented in [12, 13]. The number of locally stable states of the system is the number of local minima (or wells) in the potential. For example, a fourth-order polynomial is needed to represent a double-well potential, i.e. a system with two stable states. If the data suggests that the system that is being described by a one-well potential is slowing

changing towards a to a two-well potential (i.e. two alternative stable states), it may be interpreted as becoming increasingly sensitive to perturbations that may lead to abrupt transitions of the system between states.

This method has been applied to spatial data sets that include several snapshots of the system at a given stress level [18]. If such data sets are available across a gradient of stressor, potential analysis can be used to estimate an average stability landscape as a function of the measured environmental conditions (i.e. precipitation in the case of [18]). A changing environmental stressor may lead to changes in the landscape characteristics, such as changing from a potential with multiple minima to a potential with a single minimum. It may then be used to obtain an estimate of the environmental condition or stressor at which abrupt transitions may be expected. Here we do not test this method because our example data set are not suitable for this analysis. Nonetheless, we chose to present this method so that it can be applied to suitable datasets.

Patch-based indicators

Many ecological systems, such as shrublands in semi-arid ecosystems and mussel beds in the intertidal, exhibit striking spatial self-organized patterns [20]. It has been suggested that the nature of local ecological interactions, such as the relative scales of competition and facilitation, can strongly influence the type of emerging spatial patterns, leading to i) regular, periodic patches with a characteristic patch size [20–23], or to ii) no characteristic scale of patchiness [22–26]. These different types of spatial structures have been observed in a range of ecosystems, however their use as potential indicators of degradation has mostly been developed in the case of drylands, where both types of spatial structures exist. In drylands, it has been shown that the early warning signals that may be employed depend on the type of patchiness exhibited by the ecological system [1], as described below.

Periodic patterns: probing the shape of the patches

In ecosystems exhibiting periodic patterns, as the level of external stress increases, a spatially-homogeneous system may eventually undergo a shift to a low productivity state. The shift is preceded by an occurrence of a predictable sequence of self-organized patterns, also called ‘Turing patterns’, from gaps through labyrinths to spots. Thus, the occurrence of spotted vegetation patterns has been proposed to be an early

warning signal of imminent desertification in drylands characterized by periodic patterns [21, 27].

To characterize periodic patterns in spatial data, for instance data based on spatial imagery, one can employ different metrics derived from discrete Fourier transform (DFT). Recall from Eq (5) that DFT decomposes spatial data into periodic sine and cosine functions and determines the relative strength (also called Fourier coefficients) by which wavenumbers contribute to the spatial patterns. The values of the power spectrum I (Eq. 5) can be used to identify spatial periodicity and isotropy, i.e. whether there exists a particular orientation in the patterns.

First, the periodicity of the patterns is evaluated from the radial-spectrum (r-spectrum) obtained by summing the power spectrum at constant distances from the origin of the power spectrum, i.e. along concentric circles at a distance $r = \sqrt{p^2 + q^2}$ from the center. It quantifies the contribution of successive wavenumbers to the spatial variance of the data set. Periodic patterns are characterized by a peak in the power spectrum. The wavenumber, r_m, at which a peak occurs corresponds to the number of times that a pattern reproduces itself within a unit area of the spatial data, and therefore contains information about the scale of the pattern.

Second, isotropy (orientation) of the spatial pattern is deduced from the angular-spectrum (θ-spectrum) that is obtained by summing the values of the power spectrum using angular sectors. It quantifies the contribution of different orientations to the spatial variance of the data set. For an isotropic data set, the θ-spectrum will show uniform amplitude at all angles, whereas for an anisotropic data set, the amplitude of the spectrum will show strong amplitudes for specific orientations [6].

The power- r- and θ-spectra can be used to characterize periodic spatial patterns. For instance, Deblauwe et al. (2011) [28] applied the above mentioned techniques of DFT to periodic vegetation patterns in an extensive area of Sudan. For anisotropic, band-like patterns, with a preferred orientation (visible on the θ-spectrum), they showed that the wavenumber at which the peak occurs in the r-spectrum reduces as aridity increases (i.e. their wavelength increases as the system approaches a transition). In isotropic areas, with no preferred orientation of the pattern, the shape of the patterns shifts from gaps to labyrinths and to spots as the system becomes more degraded.

Non-periodic patterns: probing distribution of patch sizes

In contrast to regularly patterned ecosystems, there are cases where spatial processes give rise to non-periodic (irregular) patterns. In these cases, we can quantify the size of each patch and calculate the frequency of occurrence of different patch sizes. It is common practice to characterize the patchiness of these systems by a function that best describes the distribution of patch sizes. Irregular patterns may be characterized by a scale-free patch-size distribution, which means that there is no typical patch size in the ecosystem. Such a distribution may be well approximated by a pure power law [29] or by other heavy-tailed functions, such as a log-normal, a stretched exponential or a power law with cutoff [29, 30].

Scale-free distributions of vegetation patch sizes have been observed in several ecosystems [24–26, 31]. In these ecosystems, vegetation patterns characterized by a power-law distribution with an exponential cut-off at relatively large patch sizes have been hypothesized to reflect a state far from desertification. As aridity or grazing pressure increases, larger vegetation patches become fragmented into smaller ones. Computational models predict this result and show an increasing deviation from a theoretical power law as the ecosystem approaches the desertification point [24, 32]. Therefore, it has been hypothesized that an increasing deviation from power-law distribution of patch sizes can signal increasing desertification (but see [33, 34]).

One way of fitting a given distribution to data has been to use ordinary least square log-log regression, but this method is known to have substantial bias in estimating the parameter values, especially for small data sets [29,30]. Maximum likelihood methods or least-square fits of the inverse-cumulative distribution (which quantifies the number of patches whose size is larger than a given value s for different values of s) provide more accurate estimates of the parameters of most heavy-tailed functions [30].

References

1. Dakos V, Kéfi S, Rietkerk M, van Nes E, Scheffer M (2011) Slowing down in spatially patterned ecosystems at the brink of collapse. The American Naturalist 177: E153–E166.

2. Dakos V, van Nes E, Donangelo R, Fort H, Scheffer M (2010) Spatial correlation as leading indicator of catastrophic shifts. Theoretical Ecology 3: 163–174.

3. Dai L, Korolev K, Gore J (2013) Slower recovery in space before collapse of connected populations. Nature 496: 355-358.

4. Carpenter S, Brock W (2010) Early warnings of regime shifts in spatial dynamics using the discrete fourier transform. Ecosphere 1: art10.

5. Legendre P, Legendre L (1998) Numerical ecology. Amsterdam: Elsevier Science.

6. Couteron P (2002) Quantifying change in patterned semi-arid vegetation by fourier analysis of digitized aerial photographs. International Journal of Remote Sensing 23: 3407–3425.

7. Mugglestone MA, Renshaw E (1998) Detection of geological lineations on aerial photographs using two-dimensional spectral analysis. Computers and geosciences 24: 771-784.

8. Sethna JP (2007) Statistical Mechanics: Entropy, Order Parameters, and Complexity. Oxford: Oxford University Press.

9. Obórny B, Meszéna G, Szabó G (2005) Dynamics of populations on the verge of extinction. Oikos 109: 291–296.

10. Guttal V, Jayaprakash C (2009) Spatial variance and spatial skewness: leading indicators of regime shifts in spatial ecological systems. Theoretical Ecology 2: 3–12.

11. Dakos V, van Nes EH, Scheffer M (2013) Flickering as an early warning signal. Theoretical Ecology : DOI:10.1007/s12080-013-0186-4.

12. Livina V, Kwasniok F, Lenton T (2010) Potential analysis reveals changing number of climate states during the last 60 kyr. Climate of the Past 6: 77–82.

13. Livina V, Kwasniok F, Lohmann G, Kantelhardt J, Lenton T (2011) Changing climate states and stability: from pliocene to present. Climate dynamics 37: 2437–2453.

14. Guttal V, Jayaprakash C (2007) Impact of noise on bistable ecological systems. Ecological Modelling 201: 420-428.

15. Dakos V, Carpenter SR, Ellison AM, Guttal V, Ives AR, et al. (2012) Early warning signals of critical transitions: Methods for time series. PLOS ONE 7: e41010.

16. Held H, Kleinen T (2004) Detection of climate system bifurcations by degenerate fingerprinting. Geophysical Research Letters 31: L020972.

17. Livina V, Lohmann G, Mudelsee M, Lenton T (2013) Forecasting the underlying potential governing climatic time series. Physica A 392: 3891-3902.

18. Hirota M, Holmgren M, Nes EV, Scheffer M (2011) Global resilience of tropical forest and savanna to critical transitions. Science 334: 232–235.

19. Staver AC, Archibald S, Levin SA (2011) The global extent and determinants of savanna and forest as alternative biome states. Science 334: 230–232.

20. Rietkerk M, van de Koppel J (2008) Regular pattern formation in real ecosystems. Trends in Ecology & Evolution 23: 169–175.

21. Rietkerk M, Dekker SC, de Ruiter PC, van de Koppel J (2004) Self-organized patchiness and catastrophic regime shifts in ecosystems. Science 305: 1926-1929.

22. Manor A, Shnerb M (2008) Facilitation, competition, and vegetation patchiness: From scale free distribution to patterns. Journal of Theoretical Biology 253: 838–842.

23. von Hardenberg J, Kletter A, Yizhaq H, Nathan J, Meron E (2010) Periodic versus scale-free patterns in dryland vegetation. Proceedings of the Royal Society B: Biological Sciences 2077: 1771–1776.

24. Kéfi S, Rietkerk M, Alados CL, Pueyo Y, Papanastasis VP, et al. (2007) Spatial vegetation patterns and imminent desertification in mediterranean arid ecosystems. Nature 449: 213-217.

25. Scanlon T, Caylor K, Levin S, Rodriguez-Iturbe I (2007) Positive feedbacks promote power-law clustering of Kalahari vegetation. Nature 449: 209–212.

26. Foti R, del Jesus M, Rinaldo A, Rodriguez-Iturbe I (2013) Signs of critical transition in the everglades wetlands in response to climate and anthropogenic changes. Proceedings of the National Academy of Sciences 110: 6296–6300.

27. Kéfi S, Eppinga M, de Ruiter P, Rietkerk M (2010) Bistability and regular spatial patterns in arid ecosystems. Theoretical Ecology 3: 257–269.

28. Deblauwe V, Couteron P, Lejeune O, Bogaert J, Barbier N (2011) Environmental modulation of self-organized periodic vegetation patterns in sudan. Ecography 34: 990-1001.

29. Newman M (2005) Power laws, pareto distributions and zipf's law. Contemporary physics 46: 323-351.

30. White E, Enquist B, JL G (2008) On estimating the exponent of power-law frequency distributions. Ecology 89: 905–912.

31. Lin Y, Han G, Zhao M, Chang S (2010) Spatial vegetation patterns as early signs of desertification: a case study of a desert steppe in inner mongolia, china. Landscape ecology 25: 1519–1527.

32. Kéfi S, Rietkerk M, Roy M, Franc A, De Ruiter P, et al. (2011) Robust scaling in ecosystems and the meltdown of patch size distributions before extinction. Ecology letters 14: 29–35.

33. Maestre F, Escudero A (2010) Is the patch size distribution of vegetation a suitable indicator of desertification processes? reply. Ecology 91: 3742–3745.

34. Kéfi S, Alados C, Chaves R, Pueyo Y, Rietkerk M (2010) Is the patch size distribution of vegetation a suitable indicator of desertification processes? comment. Ecology 91: 3739–3742.
