# Supplementary material for: Early Warning Signals of Ecological Transitions: Methods for Spatial Patterns
Source: PLoS One. 2014 Mar 21;9(3):e92097. doi: 10.1371/journal.pone.0092097 (PMC3962379; doi:10.1371/journal.pone.0092097)
Supplement: Appendix S2 — The three models used to generate the test data sets. (PDF) [file pone.0092097.s002.pdf]

Early warning signals of ecological transitions: Methods for spatial patterns

Sonia Kéfi[1,‡*], Vishwesha Guttal[2,‡], William A. Brock[3,4], Stephen R. Carpenter[4,5], Aaron M. Ellison[6], Valerie N. Livina[7], David A. Seekell[8], Marten Scheffer[9], Egbert H. van Nes[9], Vasilis Dakos[10]

[1] Institut des Sciences de l'Evolution, CNRS, Université de Montpellier II, Montpellier, France

[2] Centre for Ecological Sciences, Indian Institute of Science, Bangalore, India

[3] Department of Economics, University of Wisconsin, Madison, Wisconsin, United States of America

[4] Department of Economics, University of Missouri, Columbia, Missouri

[5] Center for Limnology, University of Wisconsin, Madison, Wisconsin, United States of America

[6] Harvard Forest, Harvard University, Petersham, Massachusetts, United States of America

[7] National Physical Laboratory, Hampton Road, Teddington TW11 0LW, United Kingdom

[8] Department of Environmental Sciences, University of Virginia, Charlottesville, Virginia, United States of America

[9] Department of Aquatic Ecology and Water Quality Management, Wageningen University, Wageningen, The Netherlands

[10] Integrative Ecology Group, Estacion Biologica de Donana, Sevilla, Spain

‡ These authors contributed equally to this work

∗ To whom correspondence should be addressed: sonia.kefi@univ-montp2.fr

Appendix S2: The three models used to generate the test data sets

The three models we consider as same as in Dakos et al. 2011 [1]. We reproduce the equations and the table containing parameters, their description and values here.

Local positive feedback model with no patchy pattern

The first data set is based on a coupled vegetation-water dynamical model by Shnerb et al. (2003) and Guttal and Jayaprakash (2007) [2, 3]. We denote the water and biomass density at location (i, j) in a discretized two dimensional space by $w_{i,j}$ and $B_{i,j}$, respectively. Their coupled dynamics is given by

$$\begin{aligned} \frac{dw_{i,j}}{dt} &= R - w_{i,j} - \lambda w_{i,j} B_{i,j} \\ &+ D(w_{i+1,j} + w_{i-1,j} + w_{i,j+1} + w_{i,j-1} - 4w_{i,j}) + \sigma_w dW_{i,j} \qquad (1) \\ \frac{dB_{i,j}}{dt} &= \rho B_{i,j}\big(w_{i,j} - \frac{B_{i,j}}{B_c}\big) - \mu \frac{B_{i,j}}{B_{i,j} + B_0} \\ &+ D(B_{i+1,j} + B_{i-1,j} + B_{i,j+1} + B_{i,j-1} - 4B_{i,j}) + \sigma_B dW_{i,j} \qquad (2) \end{aligned}$$

Mathematically, this model is equivalent to a discretized version of coupled stochastic reaction diffusion equations. Local dynamics (also referred to as mean-field model) are based a coupled vegetation-water dynamical model [2, 3] and shows a saddle-node bifurcation as the aridity increases.

Local facilitation model, yielding scale-free patchy vegetation

The second data set was derived from a stochastic cellular automaton model with discrete spatial and time steps [4]. In this model, an ecosystem is represented by a grid of cells, each of which can be in one of three possible states: vegetated (+), empty (o) or degraded (-). Empty cells represent fertile soil whereas degraded cells represented eroded soil patches unsuitable for recolonization by vegetation. The probability of transiting from one state to the other are given by:

$$w_{[0,+]} = [\delta\rho_+ + (1 - delta)q_+](b - c\rho_+) \qquad (3)$$

$$w_{[-,0]} = r + fq_{+|-} \qquad (4)$$

$$w_{[+,0]} = m \qquad (5)$$

$$w_{[0,-]} = d \qquad (6)$$

A mean-field approximation of this model also exhibits a saddle node bifurcation as a function of

aridity [4].

Scale-dependent feedback model, yielding periodic patterns

Here, we employed a stochastic version of a three partial differential equations model describing the dynamics of vegetation biomass, soil water and surface water [5]. The equations are given by:

$$\frac{\partial O}{\partial t} = R(t) - \alpha O \frac{P + W_0 k_2}{P + k_2} + D_o \nabla^2 O + \sigma dW \quad (7)$$

$$\frac{\partial W}{\partial t} = \alpha O \frac{P + W_0 k_2}{P + k_2} - g_{max} \frac{W}{W + k_1} P - r_w W + D_w \nabla^2 W + \sigma dW \quad (8)$$

$$\frac{\partial P}{\partial t} = \left(c g_{max} \frac{W}{W + k_1} - d \right) P + D_p \nabla^2 P + \sigma dW \quad (9)$$

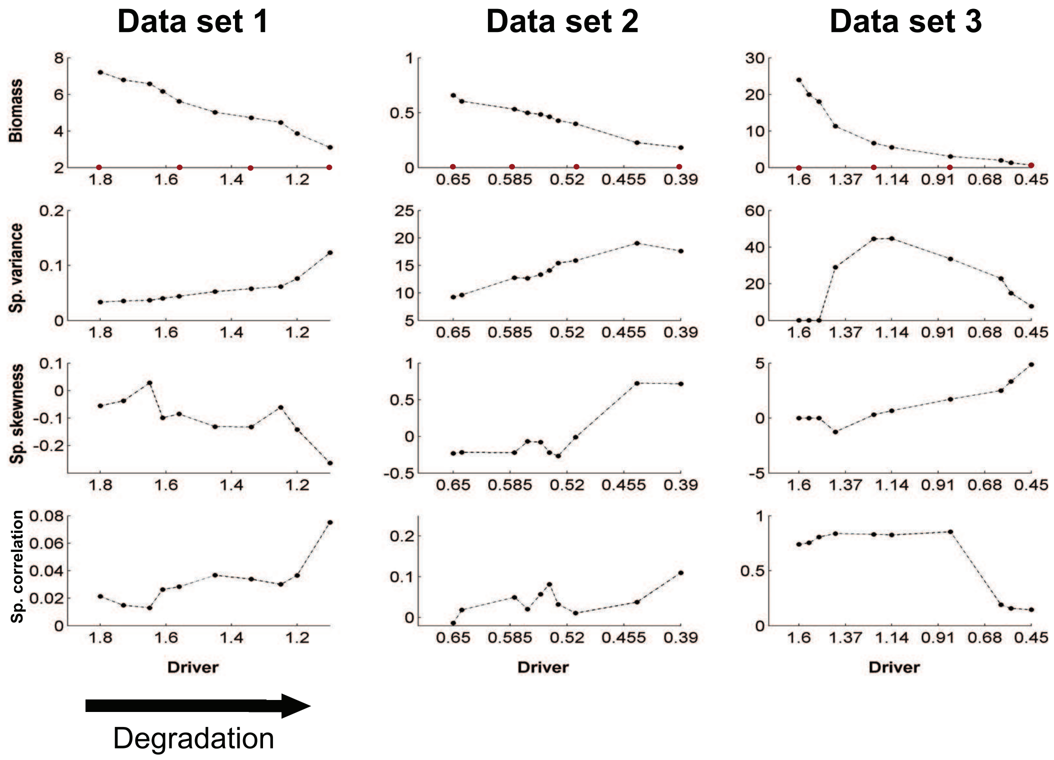

Figure S1. Generic leading indicators in the three data sets plotted as a function of the value of the driver (aridity increases from left to right along the x-axis). The black dots correspond to the ten snaphots mentioned at the end of page 13, first paragraph, as those selected. Left: local positive feedback model. Middle: local facilitation model (data transformed into quantitative data using 5x5 submatrices). Right: scale-dependent feedbcak model. First row: Biomass. Red dots along the x-axis indicate the location of the snapshots chosen for Fig. 1,3,5-7 of the main text. Second row: spatial variance. Third row: spatial skewness. Fourth row: spatial correlation between near neighbors.

Table S1. Model parameters and their values

Model parameter	Definition	Value and unit
	**Local positive feedback model**	
$w_{i,j}$	Water moisture level in each grid cell (i, j)	mm
$B_{i,j}$	Vegetation biomass in each grid cell (i, j)	g
D	Exchange (diffusion) rate	0.05 day^{-1}
λ	Water consumption rate by vegetation	0.12 $\text{g}^{-1}\text{day}^{-1}$
ρ	Maximum vegetation growth rate	day^{-1}
B_c	Vegetation carrying capacity	1 g
μ	Maximum grazing rate	2 day^{-1}
B_0	Half-saturation constant of vegetation consumption	1 g
R	Mean annual rainfall	.8-2mm day^{-1}
σ_w	Standard deviation of white noise on water moisture	0.1
σ_B	Standard deviation of white noise on vegetation biomass	0.25
$dW_{i,j}$	White noise; uncorrelated in each grid cell	0.25
	**Local facilitation model**	
$w_{[0,+]}$	Colonization probability of an unoccupied site	
$w_{[-,0]}$	Regeneration probability of a degraded site	
$w_{[,0]}$	Mortality probability of an occupied site	
$w_{[0,-]}$	Degradation probability of an unoccupied site	
ρ_+	Density of vegetated sites	
$q_{i\|j}$	Clustering vegetation intensity probability of finding a site j in state i $(+, 0, -)$	
m	Mortality probability of a vegetated site	0.1
f	Local facilitation strength; maximum effect of a neighboring vegetation site on the regeneration of a degraded site	0.9
β	Intrinsic seed production rate per vegetated site; "survival probability", "germination probability"	
ϵ	Establishment probability of seeds on 0 site in a system without competition	
b	Measures the severity of the environmental conditions $(= \beta\epsilon)$; a lower b value reflect a higher aridity level	0.3-1
δ	Fraction of seeds globally dispersed	0.1
g	Competitive effect of the global density of + sites on the establishment of new individuals	031
c	βg	0.3
r	Regeneration probability of a $-$ site without vegetated sites in its neighborhood	0.0001
d	Degradation probability of 0 sites	0.2
	**Scale-dependent feedback model**	
P	Plant density	$g\,m^{-2}$
W	Soil water	$mm\,-$
O	Surface water	$mm\,-$
c	Conversion factor for water uptake to plant biomass	5 $g\,m^{-2}\,mm^{-1}$
g_{max}	Maximum specific water uptake	0.1 $mm\,g^{-1}\,m^2 day^{-1}$
k_1	Half saturation constant of water uptake by plants	5 mm
d	Specific rate of plant density loss due to mortality	0.25 day^{-1}
α	Rate of surface water infiltration	0.4 day^{-1}
k_2	Plant density scale determining how surface water infiltration increases with P	5 $g\,m^{-2}$
W_0	Minimum surface water infiltration coefficient in the absence of plants	0.2
r_w	Soil water loss rate due to evaporation and drainage	0.4 day^{-1}
R	Rainfall	0.05-2 $mm\,day^{-1}$
D_p	Plant dispersal diffusion constant	0.01 $m^2\,day^{-1}$
D_w	Soil water diffusion constant	0.1 $m^2\,day^{-1}$
D_o	Surface water diffusion constant	100 $m^2\,day^{-1}$

References

1. Dakos V, Kéfi S, Rietkerk M, van Nes E, Scheffer M (2011) Slowing down in spatially patterned ecosystems at the brink of collapse. The American Naturalist 177: E153–E166.

2. Shnerb NM, Sarah P, Lavee H, Solomon S (2003) Reactive glass and vegetation patterns. Physical Review Letters 90: 038101.

3. Guttal V, Jayaprakash C (2007) Impact of noise on bistable ecological systems. Ecological Modelling 201: 420-428.

4. Kéfi S, Rietkerk M, van Baalen M, Loreau M (2007) Local facilitation, bistability and transitions in arid ecosystems. Theoretical Population Biology 71: 367–379.

5. Rietkerk M, Boerlijst MC, van Langevelde F, HilleRisLambers R, van de Koppel J, et al. (2002) Self-organization of vegetation in arid ecosystems. American Naturalist 160: 524-530.
