# Supplementary material for: Early Warning Signals of Ecological Transitions: Methods for Spatial Patterns
Source: PLoS One. 2014 Mar 21;9(3):e92097. doi: 10.1371/journal.pone.0092097 (PMC3962379; doi:10.1371/journal.pone.0092097)
Supplement: Appendix S3 — Results compared to the null model based on white noise. (PDF) [file pone.0092097.s003.pdf]

Early warning signals of ecological transitions: Methods for spatial patterns

Sonia Kéfi[1,‡*], Vishwesha Guttal[2,‡], William A. Brock[3,4], Stephen R. Carpenter[4,5], Aaron M. Ellison[6], Valerie N. Livina[7], David A. Seekell[8], Marten Scheffer[9], Egbert H. van Nes[9], Vasilis Dakos[10]

[1] Institut des Sciences de l'Evolution, CNRS, Université de Montpellier II, Montpellier, France

[2] Centre for Ecological Sciences, Indian Institute of Science, Bangalore, India

[3] Department of Economics, University of Wisconsin, Madison, Wisconsin, United States of America

[4] Department of Economics, University of Missouri, Columbia, Missouri

[5] Center for Limnology, University of Wisconsin, Madison, Wisconsin, United States of America

[6] Harvard Forest, Harvard University, Petersham, Massachusetts, United States of America

[7] National Physical Laboratory, Hampton Road, Teddington TW11 0LW, United Kingdom

[8] Department of Environmental Sciences, University of Virginia, Charlottesville, Virginia, United States of America

[9] Department of Aquatic Ecology and Water Quality Management, Wageningen University, Wageningen, The Netherlands

[10] Integrative Ecology Group, Estacion Biologica de Donana, Sevilla, Spain

‡ These authors contributed equally to this work

∗ To whom correspondence should be addressed: sonia.kefi@univ-montp2.fr

Appendix S3: Results compared to the null model based on white noise

All the simulations were performed for two null models: a null model based on permutation of the values of the cells of the original data set and a null model where the data sets of same size are generated by white noise with same mean and standard deviation as the original data set. The permutation results are presented in the main text. Here we show the same figures but with the white noise null model.

In the particular case of the cellular automaton model (dataset 2), we used 5x5 submatrices to calculate

coarse matrices that were 25 times smaller than the original dataset. The null matrices were calculated as follows. For the permutation null model, we did the following:

- permutation of the original matrix
- calculation of the coarse matrix on the permuted matrix
- calculation of the indicators on the coarse matrices.

For the white noise model, we did the following:

- calculation of the coarse matrix
- calculation of the mean and standard deviation of the coarse matrix
- creation of a new matrix of same dimension of the coarse matrix where the values in each cell are generated by white noise of same mean and standard deviation as the coarse matrix.

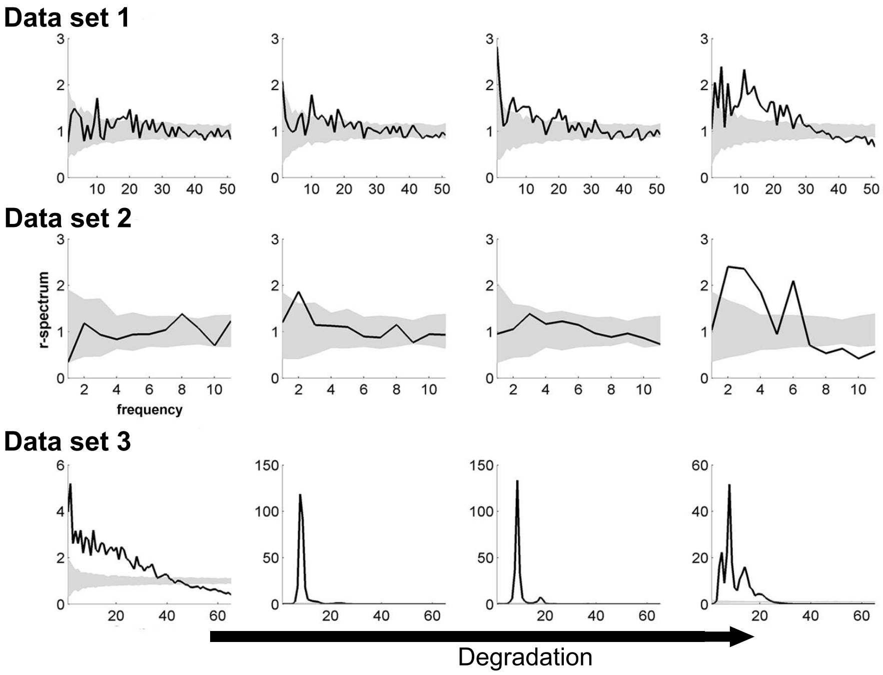

Figure S1. Radial-spectrum plotted for the three data sets along a degradation gradient. First row: local positive feedback model. Middle row: local facilitation model (original data transformed using 5x5 submatrices). Bottom row: scale-dependent feedback model. In each row, the system approaches the bifurcation point from left to right column (see Fig. S1 in Appendix S2 to visualize the location of the four snapshots along the gradient). Gray areas correspond to 95% confidence intervals obtained using 200 simulations of a white noise null model.

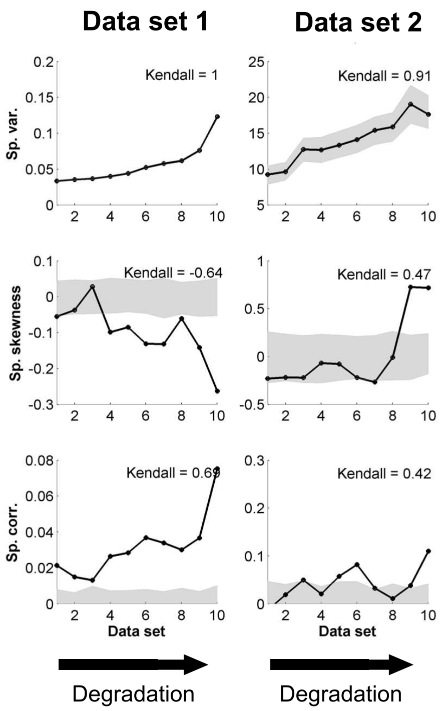

Figure S2. Generic leading indicators along a stress gradient in data sets 1 and 2. This mimicks a scenario where we would not know the exact value of the driver but where we can order the snapshots along a gradient. Within each panel, the system approaches the bifurcation point from left to right along the x-axis. Left column: local positive feedback model. Right: local facilitation model (original data transformed using 5x5 sub-matrices). First row: spatial variance. Second row: spatial skewness. Third row: spatial correlation at lag one. In each panel, Kendall's τ, quantifying the trend of the indicator, is indicated. Gray areas correspond to 95% confidence intervals obtained using 200 simulations of a white noise null model.

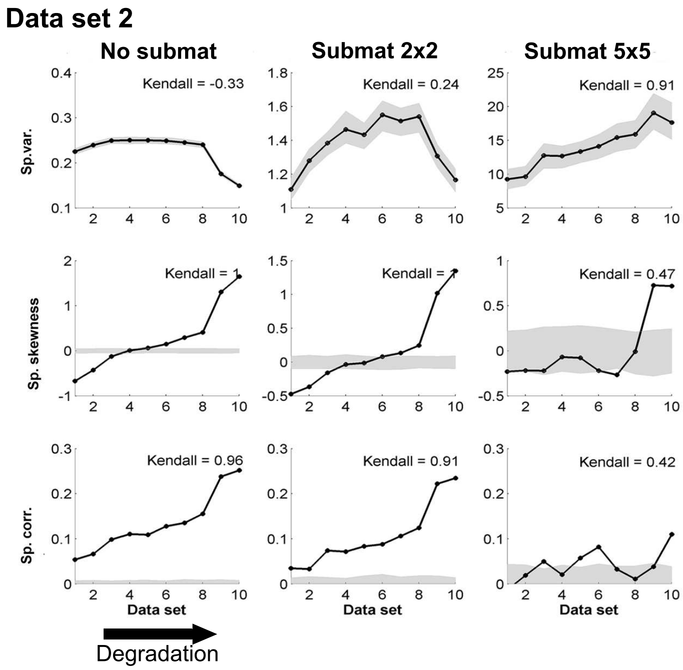

Figure S3. Generic leading indicators in data set 2 along a degradation gradient using different coarsening of the original data. In each panel, from left to right, the system approaches the bifurcation point. Left: original data. Middle: original data transformed using 2x2 sub-matrices. Middle: original data transformed using 5x5 sub-matrices. First row: spatial variance. Second row: spatial skewness. Third row: spatial correlation at lag one. In each panel, Kendall's τ quantifies the strength of the trend of the indicator. Gray areas correspond to 95% confidence intervals obtained using 200 simulations of a white noise null model.

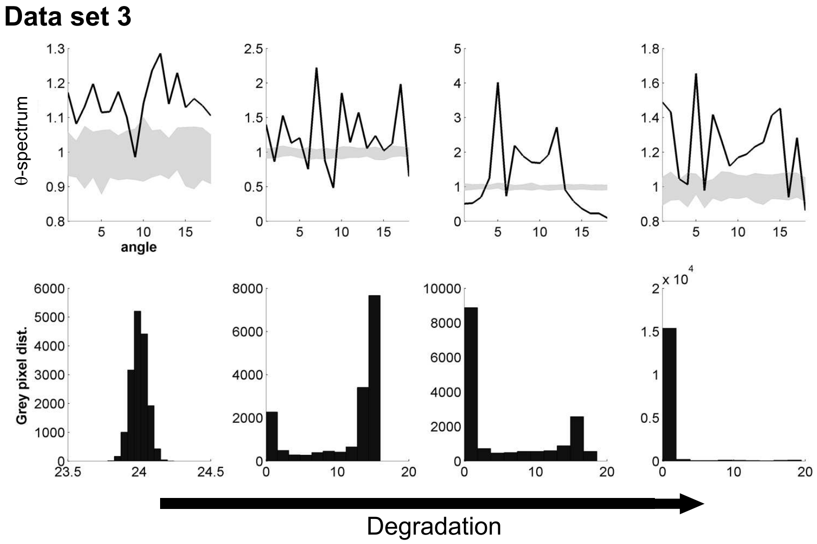

Figure S4. Analysis of the periodic patterns of data set 3, i.e. the scale-dependant feedback model. In each row, the system approaches the bifurcation point from left to right column (see Fig. S1 in Appendix S2 to visualize the location of the snapshots along the gradient). First row: θ-spectrum. Second row: distribution of pixels on the image. Gray areas corresponds to 95% confidence interval obtained using 200 simulations of a white noise null model. Second row: histogram of the values of the data set.
